# Electric Field Effects on Amine Regeneration in Post-Combustion Carbon Capture—Part II: Mode-Selective Infrared Laser Excitation Probed by Nonequilibrium Ehrenfest Electron-Nuclear Dynamics

**DOI:** 10.3390/molecules31193475

**Published:** 2026-09-29

**Authors:** Nasser D. Afify, Xianfeng Fan, Martin B. Sweatman

**Affiliations:** Institute for Materials and Processes, School of Engineering, The University of Edinburgh, Edinburgh EH9 3FB, UK; x.fan@ed.ac.uk (X.F.); martin.sweatman@ed.ac.uk (M.B.S.)

**Keywords:** post-combustion carbon capture, amine regeneration, density functional tight binding, nonequilibrium Ehrenfest electron–nuclear dynamics, infrared laser excitation, mode-selective infrared laser excitation, laser-induced heating, laser-induced nonthermal effects

## Abstract

Amine regeneration is the most energy-intensive step in amine-based post-combustion carbon capture (PCCC), and reducing its energy demand is essential for improving the overall efficiency of the capture process. In Part I of this series, static electric fields were shown to stabilize CO_2_ absorption products and increase the energy requirements for amine regeneration. In Part II, we investigate whether the fundamentally different response to time-dependent infrared laser excitation could provide molecular mechanisms for reducing the energy demand of amine regeneration. Nonequilibrium Ehrenfest electron–nuclear dynamics simulations were performed for aqueous zwitterion, carbamate, and bicarbonate systems representative of CO_2_ capture by monoethanolamine (MEA) and triethanolamine (TEA), using a benchmarked density functional tight-binding framework. Pulsed and continuous-wave infrared laser excitation was applied at frequencies corresponding to selected molecular vibrational modes. Laser-induced heating was strongly mode selective, with the C_CO_2__–O_CO_2__–C_CO_2__ bending mode consistently producing the largest overall temperature increase across all three systems. However, the modes producing the strongest total-system heating were not necessarily those that preferentially deposited vibrational kinetic energy into the CO_2_–amine species rather than the surrounding water. Preferential energy deposition was strongly system dependent, being greatest for the symmetric C_CO_2__–O_CO_2__ stretch in the zwitterion, the C_CO_2__–N_MEA_ stretch in the carbamate, and the C_CO_2__–O_OH_ stretch in the bicarbonate. Infrared laser excitation also produced pronounced mode-dependent structural and energetic perturbations of the regeneration-relevant C_CO_2__–N_MEA_ and C_CO_2__–O_OH_ bonds, including increases in their mean lengths and energies, broadening of their bond-length and bond-energy distributions, and increased probabilities of sampling transient elongated-bond configurations. Importantly, the strongest bond perturbations did not always result from direct excitation of the monitored bond, consistent with vibrational coupling and energy redistribution. These results identify three complementary effects that may contribute to infrared-assisted amine regeneration: efficient total-system heating, preferential energy deposition into the CO_2_–amine species, and mode-selective structural and energetic perturbations of bonds involved in CO_2_ retention. Because no single vibrational mode maximizes all three effects across the investigated systems, selecting an excitation frequency is inherently a multi-objective problem. The findings provide a molecular-level basis for investigating infrared-assisted regeneration strategies, although their potential to reduce process-level energy consumption remains to be established.

## 1. Introduction

Amine-based post-combustion carbon capture (PCCC) is among the most mature technologies for mitigating CO_2_ emissions [1]. However, the high energy demand associated with solvent regeneration remains a major obstacle to widespread implementation of PCCC. Regeneration is typically performed at temperatures of approximately 120–140 °C and constitutes the most energy-intensive stage of the carbon capture process [2,3,4,5,6,7]. Considerable research efforts have therefore focused on developing alternative regeneration strategies capable of reducing the thermal energy demand of conventional PCCC steam stripping [2,3,4,5,6,8,9,10,11]. Among these, electromagnetic-radiation-assisted regeneration methods, including microwave heating [2,12,13,14,15] and frequency-tuned infrared heating [16], have emerged as promising alternatives. Unlike conventional conductive heating, electromagnetic fields can couple directly to molecular dipoles, charge distributions, and vibrational modes, potentially enabling both efficient energy deposition and selective perturbation of molecular processes relevant to amine regeneration [17,18]. Such interactions may produce thermal effects through increased bulk and preferential heating [16] while also inducing nonthermal changes in molecular structure and reaction energetics.

In Part I of this series [19], we examined the effects of static electric fields on the thermodynamics and kinetics of CO_2_ absorption and regeneration reactions in aqueous and non-aqueous solutions of monoethanolamine (MEA) and triethanolamine (TEA) using density functional theory calculations. The results showed that static electric fields stabilize the CO_2_ absorption products and generally reduce the activation barriers for CO_2_ absorption. However, the same stabilization increases the activation barriers and reaction enthalpies associated with amine regeneration, indicating that static electric fields are unlikely to facilitate low-energy amine regeneration. These findings raise the question of whether time-dependent electric fields, such as those associated with infrared laser excitation, can influence amine regeneration through fundamentally different mechanisms. Unlike static fields, infrared radiation can in principle couple selectively to molecular vibrational modes and may therefore induce both thermal heating and nonthermal structural and energetic changes that may alter the stability of CO_2_ absorption products.

In Part II, we extend this investigation to the fundamentally different regime of time-dependent electric fields, which oscillate on femtosecond timescales comparable to molecular vibrational motion. Whereas static fields primarily exert a sustained influence on the thermodynamic stability of reaction intermediates, infrared laser excitation can couple directly to molecular vibrational modes and drive the system far from equilibrium. Here, time-dependent Ehrenfest electron–nuclear dynamics is employed to examine the coupled electronic and nuclear response of aqueous CO_2_–amine systems under infrared laser excitation. The principal objective is to determine whether frequency-selective infrared laser excitation of chemically relevant vibrational modes can transiently destabilize CO_2_ absorption products and promote structural changes along regeneration-relevant reaction coordinates, thereby counteracting the unfavourable static-field stabilization identified in Part I [19]. This study builds on our previous classical molecular dynamics study, which demonstrated preferential infrared heating of aqueous amine solutions at selected vibrational frequencies [16]. While that earlier work considered only classical molecular dynamics simulations and thermal energy deposition, the present study explicitly incorporates nonequilibrium electron–nuclear dynamics to examine both the thermal and nonthermal contributions to infrared-assisted amine regeneration.

Investigating the nonthermal effects induced by infrared laser excitation during amine regeneration requires a computational framework capable of describing the coupled evolution of electronic structure and nuclear motion under a time-dependent electromagnetic field. Conventional static quantum chemical methods are well suited to determining equilibrium geometries, transition states, and activation barriers, but they cannot capture the nonequilibrium response of a molecular system to a time-dependent perturbation. Ehrenfest dynamics provides an appropriate framework for this purpose [20]. In this mixed quantum–classical approach, the total molecular wavefunction is approximated as a product of electronic and nuclear components. The electronic subsystem evolves quantum mechanically in the time-dependent potential generated by the moving nuclei, while the nuclei are propagated classically under forces determined from the instantaneous electronic state, corresponding to motion on an effective time-dependent potential-energy surface. This self-consistent coupling enables direct treatment of field-induced polarization, transient charge redistribution, vibrational excitation, and the coupled evolution of electronic and nuclear degrees of freedom under nonequilibrium conditions [20].

For molecular systems of the size and complexity considered here, two complementary implementations of Ehrenfest dynamics are particularly relevant. At the higher-accuracy level, time-dependent density functional theory (TD-DFT)-based Ehrenfest dynamics provides an ab initio description of coupled electron–nuclear evolution by propagating time-dependent Kohn–Sham orbitals in the presence of an external field, thereby enabling the treatment of electronic polarization, charge redistribution, and field-induced structural changes [21]. However, the substantial computational cost of real-time TD-DFT generally restricts its application to relatively small systems and very short simulation times. Real-time density functional tight binding (TD-DFTB) offers a computationally more efficient alternative that retains the essential description of coupled electronic and nuclear dynamics while substantially reducing the computational expense [22,23]. This efficiency makes TD-DFTB particularly suitable for larger molecular systems containing explicit solvent and for simulations extending into the picosecond regime, where full TD-DFT propagation would be computationally prohibitive.

Semiempirical computational chemistry methods occupy an intermediate position between high-accuracy ab initio electronic-structure approaches and computationally inexpensive classical force fields. Their principal aim is to reduce the cost of electronic-structure calculations through controlled approximations combined with empirical parameterization against experimental data or higher-level quantum chemical results. One major class of semiempirical methods is based on the zero-differential-overlap (ZDO) approximation, including CNDO (Complete Neglect of Differential Overlap), INDO (Intermediate Neglect of Differential Overlap), and NDDO (Neglect of Diatomic Differential Overlap). These methods simplify the treatment of electron–electron interactions by neglecting or approximating selected classes of overlap integrals [24]. Building on the NDDO framework, widely used Hamiltonians such as MNDO, AM1, PM3, PM6, and PM7 were subsequently developed, with parameters optimized to reproduce thermochemical properties, molecular geometries, and selected electronic characteristics [24].

A second major class of semiempirical computational methods comprises tight-binding approaches, most notably density functional tight binding (DFTB). In DFTB, the Kohn–Sham density functional theory total energy is expanded as a Taylor series about a reference electron density and truncated at low order, yielding a computationally efficient formulation that retains a close formal connection to DFT [25]. More recently, extended tight-binding (xTB) methods have emerged as related approaches incorporating improved treatments of electrostatics and dispersion together with broad parameterization across the periodic table [25]. Semiempirical methods may therefore be broadly classified into ZDO/NDDO-based approaches, DFTB-type methods, and modern extended tight-binding variants such as xTB. These families of methods differ primarily in their underlying approximations, functional forms, and parameterization strategies rather than constituting a strict hierarchy of predictive accuracy.

Within the DFTB+ code framework [26], the semiempirical computational chemistry approaches most relevant to the present study include the MIO, 3OB, OB2, and 3OB-FREQ DFTB parameter sets, together with the GFN1-xTB and GFN2-xTB methods. MIO is one of the earlier self-consistent-charge DFTB parameterizations and was developed primarily for organic and biomolecular systems containing H, C, N, O, P, and S; it has therefore been widely applied in geometry optimization and molecular dynamics simulations [24]. The 3OB parameter set is based on the DFTB3 formalism [26], which incorporates third-order charge-fluctuation corrections and provides an improved description of hydrogen-bonded interactions, making it particularly suitable for biomolecular and hydrogen-bonded systems [26]. OB2 is an alternative DFTB3-based parameterization developed for organic and biological molecules, with particular emphasis on improving geometrical accuracy and noncovalent interactions [26]. The 3OB-FREQ variant further refines 3OB for the prediction of equilibrium structures and vibrational frequencies, making it especially relevant to the frequency-tuned infrared laser excitation investigated in the present work.

Extended tight-binding methods provide a complementary framework with broader chemical applicability. GFN1-xTB [27] is extensively parameterized to deliver reliable predictions of molecular geometries, vibrational frequencies, and noncovalent interactions across a wide range of chemical systems. Its successor, GFN2-xTB [28], introduces several methodological advances, including multipole electrostatics and density-dependent dispersion corrections, which improve the description of hydrogen bonding, polarization, and long-range intermolecular interactions. In particular, the inclusion of anisotropic electrostatic interactions and self-consistent dispersion contributions enhances the treatment of noncovalent interactions in complex molecular environments [28].

Because semiempirical computational chemistry methods rely on approximations and empirical parameterization, their predictive accuracy can vary substantially with both the system under study and the property of interest. Benchmarking against higher-level electronic-structure methods is therefore essential before applying them to a new class of problems. For infrared laser-driven electron–nuclear dynamics simulations, accurate prediction of equilibrium geometries, including bond lengths and angles, and vibrational frequencies is particularly important because these properties directly determine the structural response and resonant coupling of the molecular system under external infrared laser excitation. Accordingly, a systematic benchmarking study was conducted to assess the performance of the MIO, 3OB, OB2, 3OB-FREQ, GFN1-xTB, and GFN2-xTB methods against density functional theory reference calculations. The aim was to identify the semiempirical approach that provides the most reliable description of the structural and vibrational properties of the studied aqueous CO_2_–amine systems while retaining the computational efficiency required for time-dependent Ehrenfest electron–nuclear dynamics simulations. The benchmarking procedure and resulting method selection are presented in Section 3 and form the basis of all subsequent infrared laser-driven simulations reported in this study.

## 2. Results and Discussion

Infrared laser excitation can influence aqueous CO_2_–amine systems through both thermal and nonthermal mechanisms. Absorption of vibrational energy can increase molecular kinetic energy and produce laser-induced heating, thereby contributing to amine regeneration through conventional thermal activation. At the same time, resonant excitation of selected vibrational modes can directly perturb chemically relevant bonds and induce structural and energetic responses that are not attributable solely to an increase in temperature. Distinguishing between these contributions is therefore essential for determining whether infrared laser excitation can provide advantages beyond simple heating and whether mode-selective excitation can influence the stability of CO_2_ absorption products in ways that are directly relevant to amine regeneration.

To address these questions, the results are presented in three subsections. Section 2.1 examines the thermal response of the zwitterion–water, carbamate–water, and bicarbonate–water systems under pulsed and continuous-wave infrared laser excitation by analysing their temperature evolution, laser-induced heating, and vibrational kinetic energy distribution. Section 2.2 focuses on laser-induced nonthermal structural and energetic effects. Finally, Section 2.3 integrates the thermal and nonthermal results to assess the role of vibrational mode selectivity in amine regeneration and to identify the excitation modes that most effectively destabilize the CO_2_ absorption products.

### 2.1. Infrared Laser Thermal Effects

**Figure 1:**
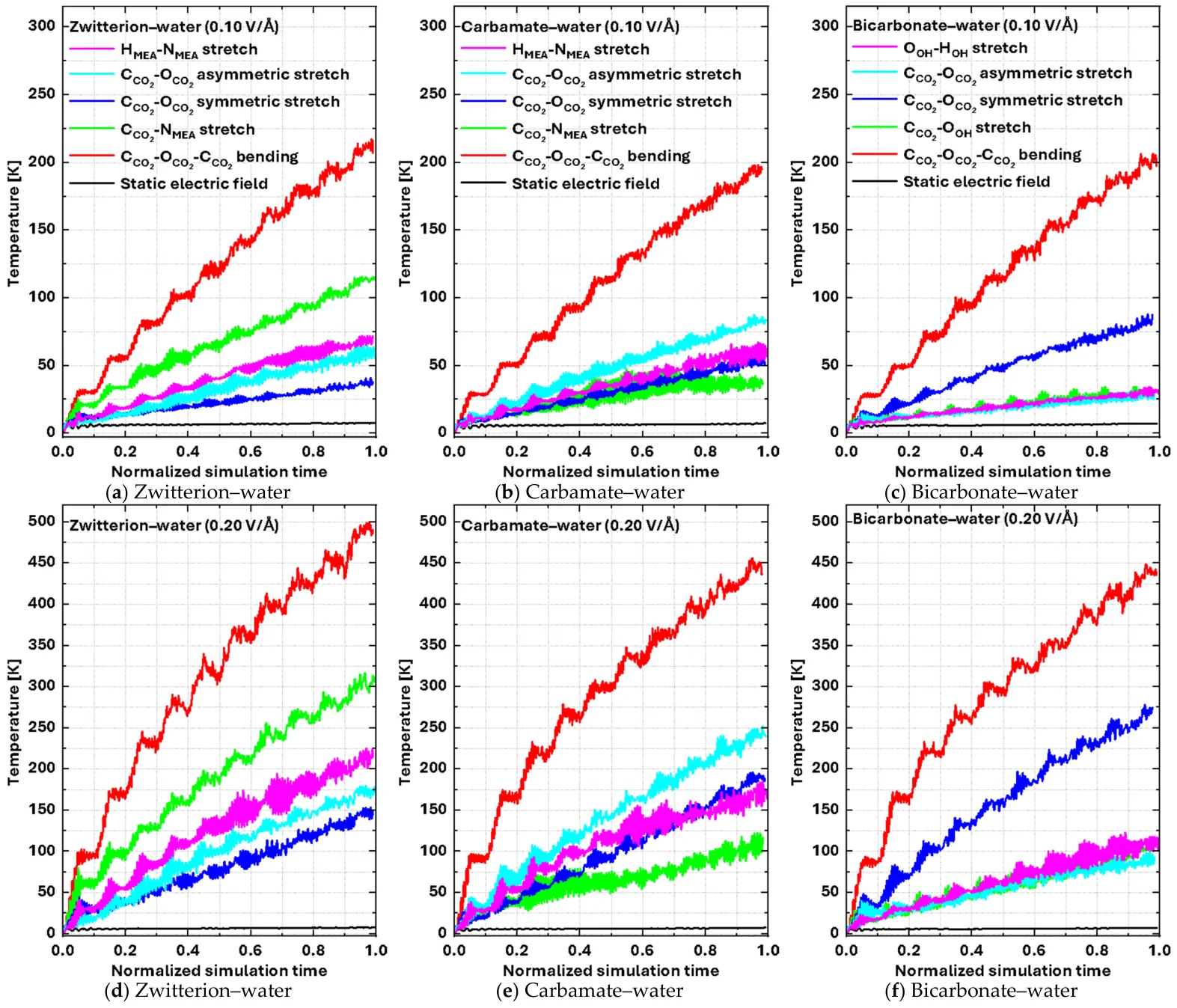
Temperature evolution of the zwitterion–water (**a**,**d**), carbamate–water (**b**,**e**), and bicarbonate–water (**c**,**f**) systems during nonequilibrium Ehrenfest electron–nuclear dynamics simulations under pulsed infrared laser excitation initiated at 0 K. Panels (**a**–**c**) correspond to an electric-field amplitude of 0.10 V/Å, while panels (**d**–**f**) correspond to 0.20 V/Å. The excitation frequencies correspond to the selected vibrational modes described in Section 3.2, and a static electric field is included as a reference. Each infrared pulse comprises three electric-field oscillation cycles followed by an equal field-free relaxation interval, with a total of 10 pulses applied during each simulation. The temperature profiles are averaged over ten independent trajectories, and the simulation time is normalized by the total duration of the complete excitation sequence.

**Figure 2:**
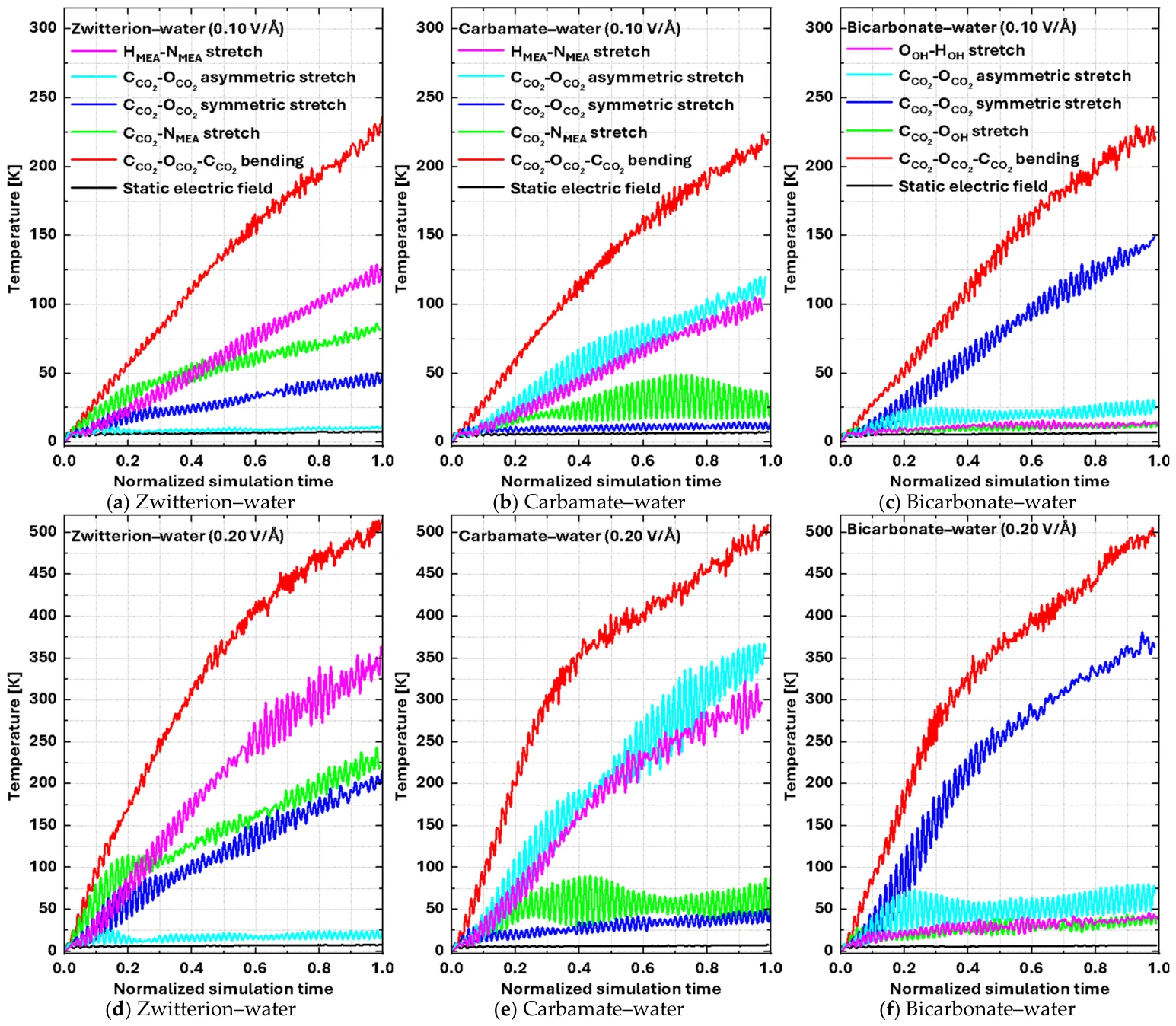
Temperature evolution of the zwitterion–water (**a**,**d**), carbamate–water (**b**,**e**), and bicarbonate–water (**c**,**f**) systems during nonequilibrium Ehrenfest electron–nuclear dynamics simulations under continuous-wave (CW) infrared laser excitation initiated at 0 K. Panels (**a**–**c**) correspond to an electric-field amplitude of 0.10 V/Å, while panels (**d**–**f**) correspond to 0.20 V/Å. The excitation frequencies correspond to the selected vibrational modes described in Section 3.2, and a static electric field is included as a reference. CW excitation comprises 30 continuous electric-field oscillation cycles applied throughout the simulation. The temperature profiles are averaged over ten independent trajectories, and the simulation time is normalized by the total simulation duration.

The thermal response of the zwitterion–water, carbamate–water, and bicarbonate–water systems under infrared laser excitation is examined first through the temperature profiles obtained from the time-dependent Ehrenfest electron–nuclear dynamics simulations. Figure 1 and Figure 2 present the temperature evolution under pulsed and continuous-wave (CW) excitation, respectively, at representative electric-field amplitudes of 0.10 and 0.20 V/Å. All simulations were initiated at 0 K, allowing the temperature increase to be attributed directly to the applied field. The applied frequencies correspond to the characteristic vibrational modes described in Section 3.2, enabling direct comparison of the heating efficiencies associated with different mode-selective excitations. A static electric field is included as a non-oscillatory reference and to facilitate comparison with the static-field behaviour reported in Part I [19]. Explicit microwave excitation was not considered because the substantially longer oscillation periods associated with microwave frequencies are incompatible with the picosecond timescales accessible to the present simulations. Figure 1 and Figure 2 therefore provide a direct comparison of how vibrational mode, laser field amplitude, and temporal excitation protocol influence infrared laser-induced heating of the three aqueous CO_2_–amine systems.

In the present nonequilibrium simulations, temperature refers to the kinetic temperature derived from the nuclear kinetic energy rather than an equilibrium thermodynamic temperature. Under resonant infrared excitation, the nuclear kinetic energy may be distributed unevenly among molecular vibrational modes rather than being fully redistributed throughout the system. The reported temperature therefore provides a measure of the overall nuclear kinetic-energy response, enabling consistent comparisons across vibrational excitation modes and laser electric-field amplitudes.

For pulsed infrared laser excitation, Figure 1 shows a clear increase in the average temperature of all three systems with simulation time, with the magnitude of heating depending strongly on both the excited vibrational mode and the applied electric-field amplitude. Increasing the field amplitude from 0.10 to 0.20 V/Å substantially enhances the temperature rise for all effective excitation modes, while preserving a pronounced mode selectivity. Most notably, excitation of the C_CO_2__–O_CO_2__–C_CO_2__ bending mode produces the strongest heating response in the zwitterion–water, carbamate–water, and bicarbonate–water systems at both field amplitudes. The secondary heating channels are system dependent: the C_CO_2__–N_MEA_ stretch is particularly effective for the zwitterion–water system, the asymmetric C_CO_2__–O_CO_2__ stretch for the carbamate–water system, and the symmetric C_CO_2__–O_CO_2__ stretch for the bicarbonate–water system. The remaining stretching modes generally produce smaller temperature increases. In contrast, the static electric field produces essentially no systematic heating. These results demonstrate that energy deposition under pulsed infrared excitation is strongly mode selective and depends sensitively on the coupling between the applied field and the vibrational dynamics of each CO_2_–amine system.

For CW infrared laser excitation, Figure 2 shows a similarly strong dependence of the thermal response on both vibrational mode and electric-field amplitude, but with a temporal behaviour distinct from that observed under pulsed excitation. In all three systems, the temperature generally increases continuously throughout the simulation, reflecting sustained energy deposition by the applied field. The C_CO_2__–O_CO_2__–C_CO_2__ bending mode again produces the largest temperature increase at both 0.10 and 0.20 V/Å. However, several secondary modes also exhibit substantial heating under CW excitation. In the zwitterion–water system, the H_MEA_–N_MEA_ stretching mode becomes the second most effective heating channel, particularly at 0.20 V/Å. In the carbamate–water system, excitation of the asymmetric C_CO_2__–O_CO_2__ stretching mode produces a thermal response approaching that of the bending mode, while in the bicarbonate–water system the symmetric C_CO_2__–O_CO_2__ stretching mode is clearly the second most effective. The static electric field again produces no systematic temperature increase. These results demonstrate that sustained resonant excitation preserves strong mode selectivity while allowing selected stretching modes to contribute substantially to the overall thermal response.

**Figure 3:**
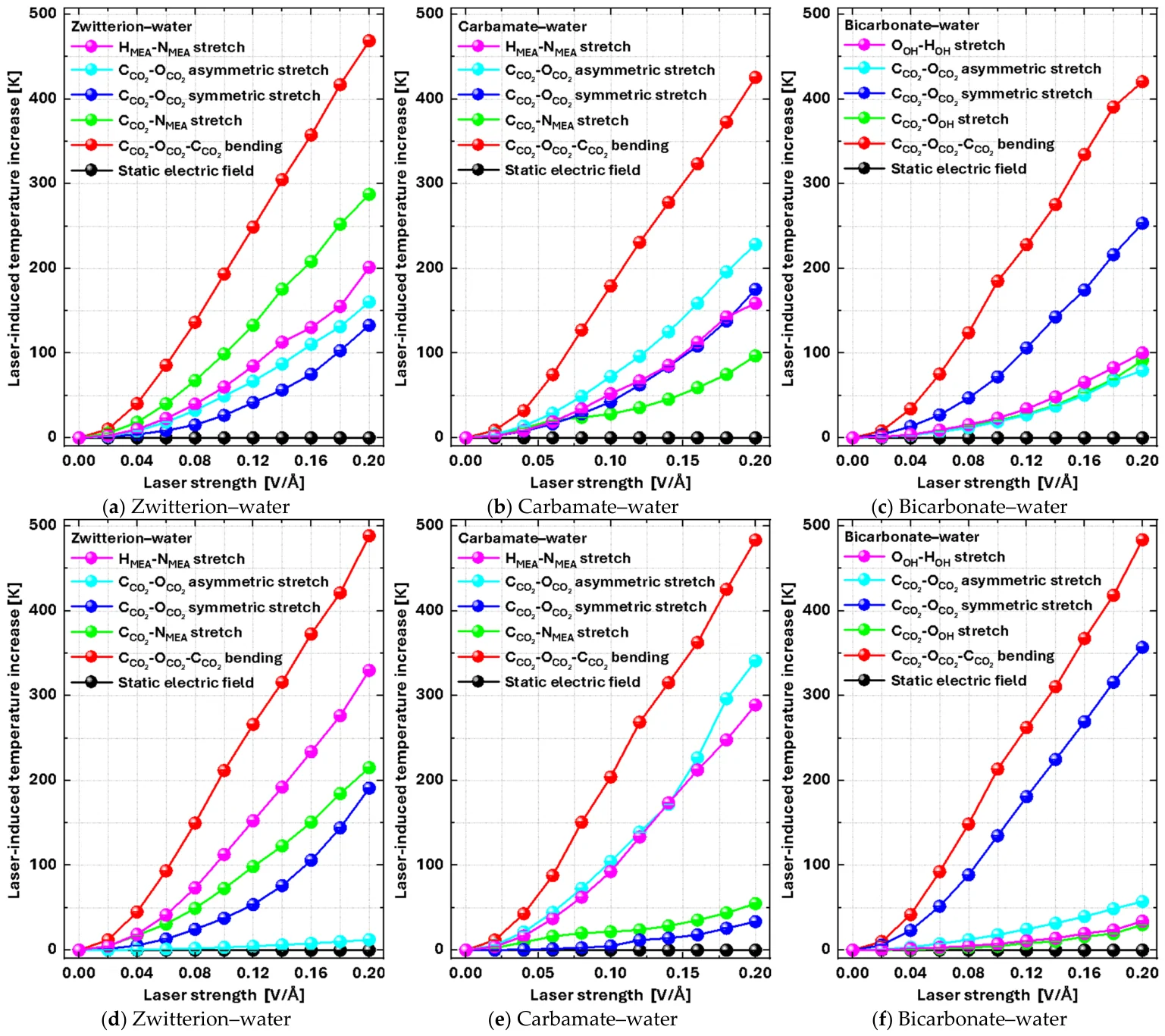
Laser-induced temperature increase, ΔT, as a function of infrared laser electric-field amplitude for the zwitterion–water (**a**,**d**), carbamate–water (**b**,**e**), and bicarbonate–water (**c**,**f**) systems under pulsed (**a**–**c**) and continuous-wave (CW) (**d**–**f**) excitation initiated at 0 K. ΔT was calculated as the difference between the average temperature obtained under laser excitation and that from the corresponding zero-field simulation. For both the pulsed and CW simulations, the averaging interval comprised the final three electric-field oscillation cycles, corresponding to the final pulse of the pulsed excitation protocol. The excitation frequencies correspond to the selected vibrational modes described in Section 3.2, and a static electric field is included as a reference. Error bars represent the standard deviation over the ten independent trajectories.

To quantify the heating response more directly, Figure 3 presents the net laser-induced temperature increase, ΔT, as a function of the applied electric-field amplitude for both pulsed and CW excitation. For each excitation condition, ΔT was calculated relative to the corresponding zero-field simulation, thereby isolating the temperature increase induced by the external infrared field. For both the pulsed and CW simulations, the average temperature was evaluated over the final three electric-field oscillation cycles, corresponding to the final pulse in the pulsed simulations. This analysis provides a consistent basis for comparing the heating efficiencies of the different vibrational modes, field amplitudes, and excitation protocols across the three CO_2_–amine systems.

The pulsed-excitation results in Figure 3a–c confirm the pronounced vibrational mode selectivity observed in the temperature profiles. The C_CO_2__–O_CO_2__–C_CO_2__ bending mode is consistently the most efficient heating channel across all three systems and over nearly the entire field-strength range, producing ΔT values of approximately 420–465 K at 0.20 V/Å. The relative importance of the remaining modes is strongly system dependent. In the zwitterion–water system, the C_CO_2__–N_MEA_ stretch provides the second-largest temperature increase, reaching approximately 285 K at 0.20 V/Å. In the carbamate–water system, the asymmetric C_CO_2__–O_CO_2__ stretch is the strongest secondary heating channel, producing ΔT of approximately 225 K, whereas in the bicarbonate–water system the symmetric C_CO_2__–O_CO_2__ stretch is the second most effective, reaching approximately 250 K. The other stretching modes produce smaller, although still measurable, temperature increases, particularly at the higher field amplitudes. By contrast, the static electric field produces essentially no net heating over the investigated field-strength range. These results demonstrate that pulsed infrared heating is governed not simply by the applied field amplitude, but also by the specific vibrational mode through which energy is introduced into each CO_2_–amine system.

The CW results in Figure 3d–f show that continuous excitation alters the relative heating efficiencies of several secondary vibrational modes while preserving the C_CO_2__–O_CO_2__–C_CO_2__ bending mode as the dominant heating channel. At 0.20 V/Å, excitation of this bending mode produces ΔT values of approximately 480–490 K across the three systems. In the zwitterion–water system, the H_MEA_–N_MEA_ stretching mode becomes particularly effective under CW excitation, reaching a temperature increase of about 325 K and substantially exceeding the response of the same mode under pulsed excitation. In the carbamate–water system, the asymmetric C_CO_2__–O_CO_2__ stretching mode becomes the strongest secondary channel, producing a ΔT of approximately 340 K. For bicarbonate–water, the symmetric C_CO_2__–O_CO_2__ stretching mode is clearly the second most effective excitation, reaching approximately 355 K at the highest field amplitude. These results show that sustained resonant driving can substantially enhance energy accumulation through selected stretching modes, although the CO_2_-centred bending vibration remains the most consistently effective heating pathway across all three systems.

A further feature of Figure 3 is the nonlinear dependence of ΔT on the applied electric-field amplitude. For most vibrational modes, the temperature increase is relatively small at low field amplitudes but rises progressively more rapidly as the field strength increases, with the curvature being particularly pronounced for the most strongly coupled modes. This behaviour is evident for the C_CO_2__–O_CO_2__–C_CO_2__ bending mode in all three systems and for several secondary modes under CW excitation, such as the H_MEA_–N_MEA_ stretch in zwitterion–water, the asymmetric C_CO_2__–O_CO_2__ stretch in carbamate–water, and the symmetric C_CO_2__–O_CO_2__ stretch in bicarbonate–water. The results therefore indicate that the efficiency of laser energy deposition is not simply proportional to the applied field amplitude, but instead reflects a strongly mode-dependent response that becomes increasingly pronounced at higher field strengths. Given the picosecond timescales accessible to the present simulations, the use of elevated field amplitudes provides a practical means of resolving these nonlinear, mode-selective heating trends within computationally manageable timescales.

**Figure 4:**
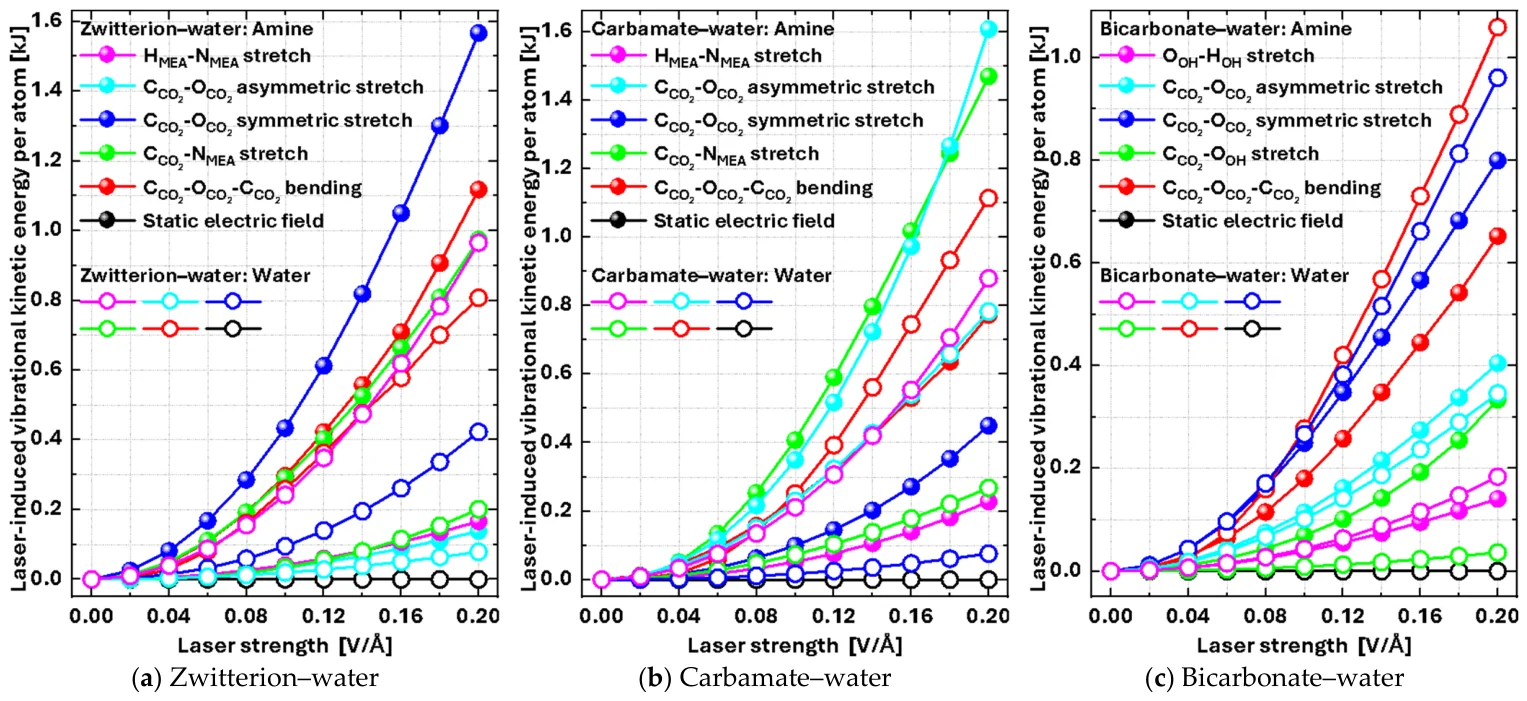

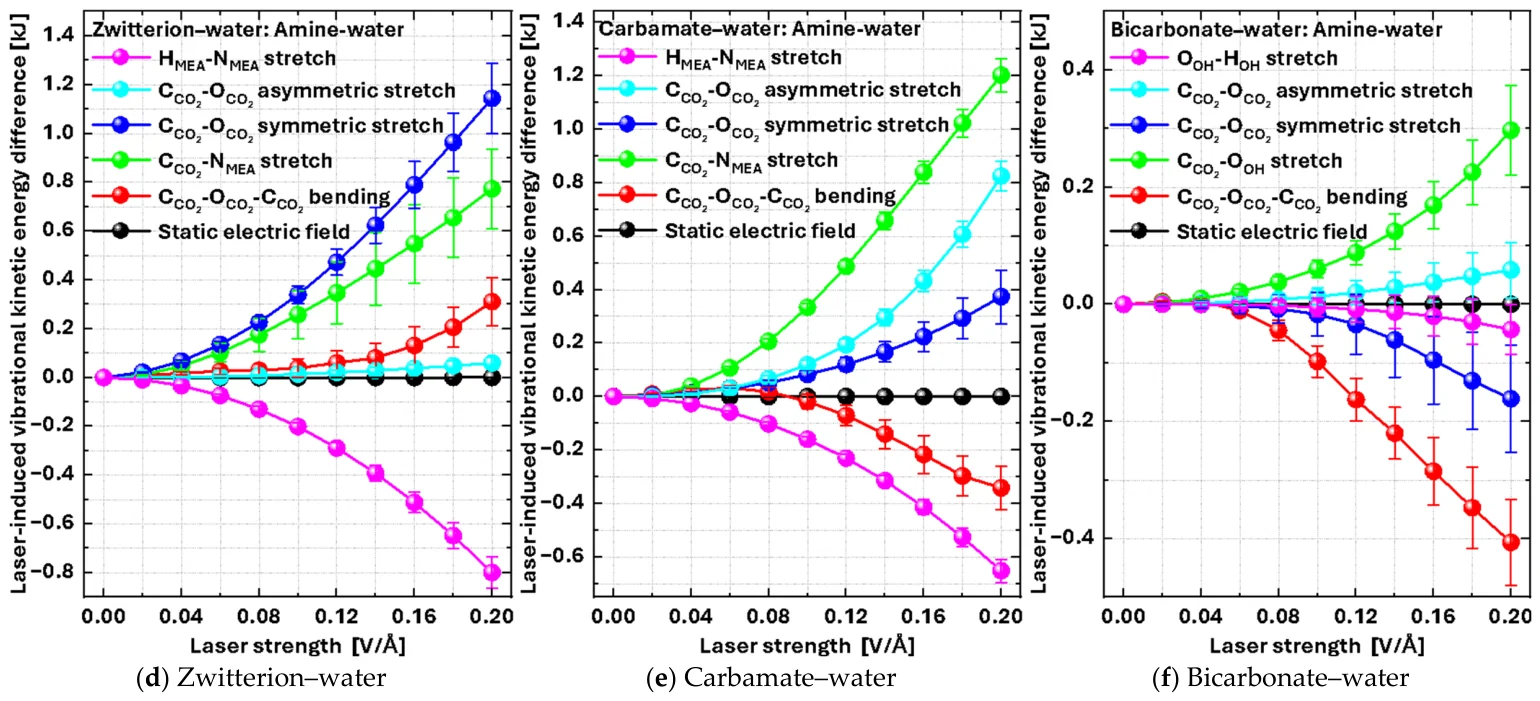
Partitioning of laser-induced vibrational kinetic energy between the CO_2_–amine species and the surrounding water during CW infrared laser excitation initiated at 0 K. Panels (**a**–**c**) show the laser-induced vibrational kinetic energy per atom of the CO_2_–amine species (filled symbols) and water molecules (open symbols) for the zwitterion–water, carbamate–water, and bicarbonate–water systems, respectively. Panels (**d**–**f**) show the corresponding difference between the vibrational kinetic energies of the CO_2_–amine species and water, providing a measure of preferential energy deposition into either subsystem. Positive values indicate preferential energy deposition into the CO_2_–amine species, whereas negative values indicate preferential energy deposition into the surrounding water. The results were obtained by analysing the first 12 electric-field oscillation cycles of the CW simulations. The excitation frequencies correspond to the selected vibrational modes described in Section 3.2, and a static electric field is included as a reference. Results are averaged over ten independent trajectories; error bars represent the corresponding standard deviations.

Although Figure 1, Figure 2 and Figure 3 establish the strongly mode-selective nature of infrared laser heating, the total-system temperature does not reveal how the absorbed vibrational energy is partitioned between the CO_2_–amine species and the surrounding water. This distinction is important because preferential deposition of energy into the CO_2_–amine species, rather than into the solvent, could enhance the effectiveness of infrared laser excitation for regeneration. To examine this energy partitioning directly, the CW simulations were analysed separately for the CO_2_–amine species and the surrounding water molecules. The resulting mode- and field-dependent vibrational kinetic energies are presented in Figure 4. In this figure, panels (a–c) compare the laser-induced vibrational kinetic energy per atom of the CO_2_–amine species (filled symbols) with that of the surrounding water (open symbols), while panels (d–f) show the corresponding vibrational kinetic energy difference, where positive and negative values indicate preferential energy deposition into the CO_2_–amine species and water, respectively.

For the zwitterion–water system (Figure 4a,d), excitation of the symmetric C_CO_2__–O_CO_2__ stretching mode produces by far the strongest preferential deposition of vibrational kinetic energy into the zwitterion relative to the surrounding water, with the energy difference increasing markedly with field strength. Excitation of the C_CO_2__–N_MEA_ stretching mode also preferentially energizes the zwitterion, although to a lesser extent, while the C_CO_2__–O_CO_2__–C_CO_2__ bending and asymmetric C_CO_2__–O_CO_2__ stretching modes produce substantially smaller positive differences. In contrast, excitation of the H_MEA_–N_MEA_ stretching mode increasingly favours energy deposition into the surrounding water as the field strength increases, leading to a pronounced negative zwitterion–water energy difference. These results demonstrate that the magnitude of laser-induced vibrational energy absorption and the extent to which that energy is preferentially localized within the CO_2_–amine species are distinct characteristics of the excited vibrational mode.

For the carbamate–water system (Figure 4b,e), excitation of the C_CO_2__–N_MEA_ stretching mode produces the strongest preferential deposition of vibrational kinetic energy into the carbamate-containing species, with the carbamate–water energy difference increasing substantially with field strength. Excitation of the asymmetric C_CO_2__–O_CO_2__ stretching mode also strongly favours energy localization within the carbamate species, although to a lesser extent, while the symmetric C_CO_2__–O_CO_2__ stretching mode produces a smaller positive difference. In contrast, excitation of the C_CO_2__–O_CO_2__–C_CO_2__ bending mode preferentially deposits energy into the surrounding water at higher field strengths, whereas the H_MEA_–N_MEA_ stretching mode shows the strongest preference for solvent excitation and produces the largest negative carbamate–water energy difference. These results further demonstrate that the vibrational modes producing substantial energy absorption by the CO_2_–amine species are not necessarily those that most selectively localize the absorbed energy within it relative to the surrounding solvent.

For the bicarbonate–water system (Figure 4c,f), the partitioning of laser-induced vibrational energy differs markedly from that observed for the MEA-based systems. Excitation of the C_CO_2__–O_OH_ stretching mode produces the strongest preferential localization of vibrational kinetic energy within the bicarbonate-containing species, with the bicarbonate–water energy difference increasing steadily with field strength. The asymmetric C_CO_2__–O_CO_2__ stretching mode also preferentially heats the bicarbonate species, although to a lesser extent, whereas the O_OH_–H_OH_ stretching mode shows a small negative difference, indicating a slight preference for energy deposition into the surrounding water. In contrast, excitation of the symmetric C_CO_2__–O_CO_2__ stretching mode preferentially deposits energy into the surrounding water, and this effect is even more pronounced for the C_CO_2__–O_CO_2__–C_CO_2__ bending mode, which produces the largest negative bicarbonate–water energy difference at high field strengths. These results further emphasize that strong total vibrational energy absorption does not necessarily correspond to preferential localization of that energy within the CO_2_–amine absorption product.

Figure 4 demonstrates that preferential localization of laser-induced vibrational energy within the CO_2_–amine species is strongly system and mode dependent. For the zwitterion–water system, the symmetric C_CO_2__–O_CO_2__ stretching mode produces the strongest preferential excitation of the zwitterion, whereas direct excitation of the C_CO_2__–N_MEA_ stretch is most effective for the carbamate–water system and excitation of the C_CO_2__–O_OH_ stretch is most effective for the bicarbonate–water system. Conversely, several modes that produce substantial total-system heating preferentially deposit energy into the surrounding water; most notably, the C_CO_2__–O_CO_2__–C_CO_2__ bending mode favours solvent heating in the carbamate–water and bicarbonate–water systems despite being the most effective overall heating mode identified in Figure 1, Figure 2 and Figure 3. These results demonstrate that efficient absorption of infrared laser energy and preferential localization of that energy within the CO_2_–amine absorption product are distinct characteristics. Consequently, both should be considered when identifying vibrational modes that may be advantageous for infrared-assisted amine regeneration.

In summary, the thermal effects presented in Figure 1, Figure 2, Figure 3 and Figure 4 demonstrate that the response of aqueous CO_2_–amine systems to infrared laser excitation is strongly mode selective and depends on the excitation protocol, field amplitude, and identity of the CO_2_ absorption product. The C_CO_2__–O_CO_2__–C_CO_2__ bending mode provides the most consistently effective pathway for total-system heating under both pulsed and CW excitation, whereas sustained CW driving substantially enhances the thermal response of several secondary stretching modes. Analysis of the laser-induced vibrational energy distribution further reveals that the modes responsible for the strongest overall heating are not necessarily those that preferentially heat the CO_2_–amine species. Instead, preferential energy localization is system dependent, with the symmetric C_CO_2__–O_CO_2__ stretch favoured for preferentially heating the zwitterion, the C_CO_2__–N_MEA_ stretch for the carbamate, and the C_CO_2__–O_OH_ stretch for the bicarbonate. Thus, total laser energy absorption and selective localization of vibrational energy within the reactive species represent distinct aspects of the infrared laser response. While these results establish the thermal behaviour and energy partitioning of the three systems, they do not determine whether infrared laser vibrational excitation can also directly perturb the molecular bonds responsible for retaining CO_2_. The following section therefore examines the corresponding nonthermal structural and energetic responses of the CO_2_–amine absorption products.

### 2.2. Infrared Laser Nonthermal Effects

**Figure 5:**
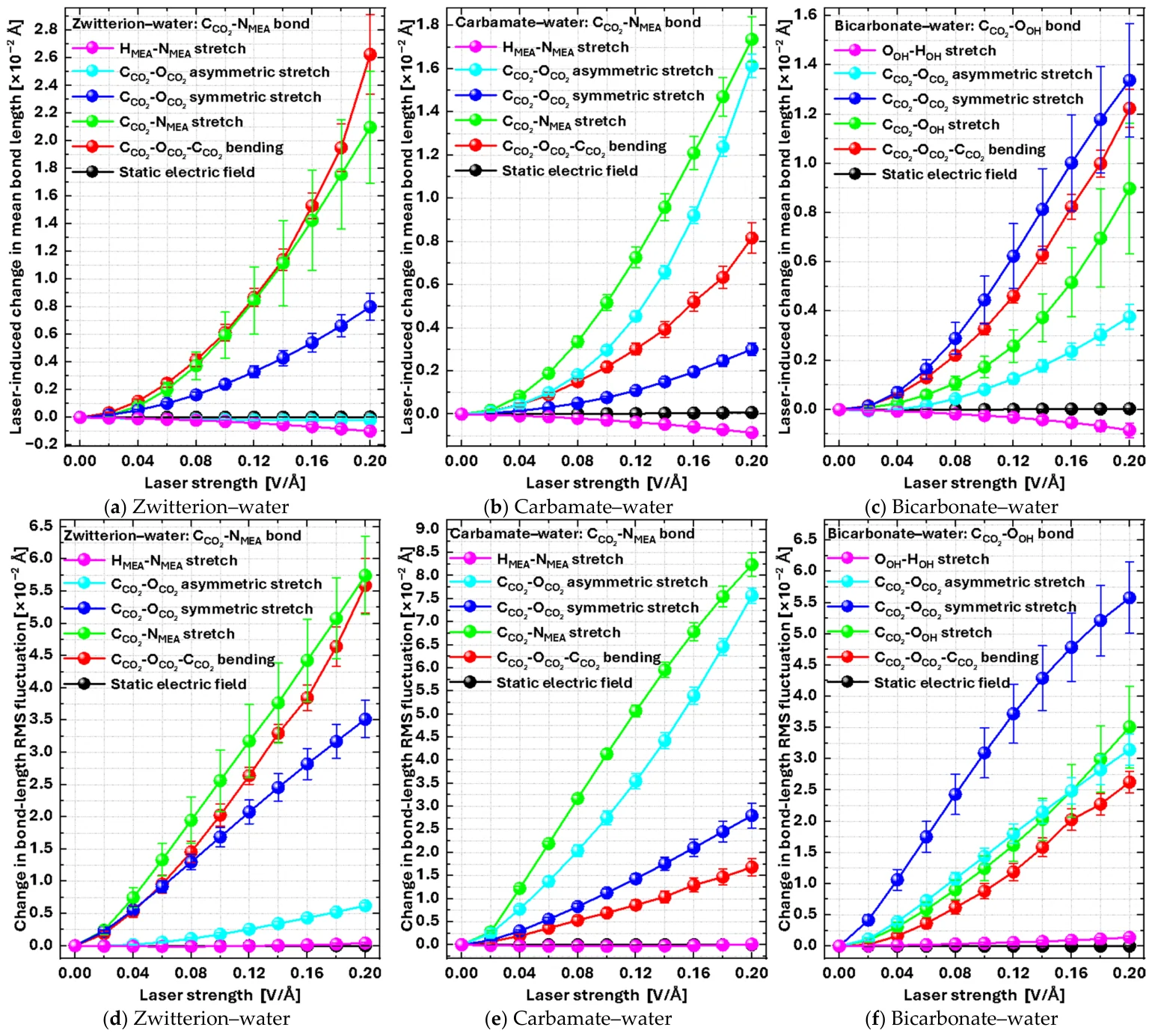
Nonthermal structural response of the aqueous zwitterion–water (**a**,**d**), carbamate–water (**b**,**e**), and bicarbonate–water (**c**,**f**) systems under CW infrared laser excitation initiated at 0 K. Panels (**a**–**c**) show the laser-induced change in the mean length of the regeneration-relevant bond, corresponding to the C_CO_2__–N_MEA_ bond for the zwitterion and carbamate systems and the C_CO_2__–O_OH_ bond for the bicarbonate system. Panels (**d**–**f**) show the corresponding change in the root-mean-square (RMS) bond-length fluctuation, calculated about the mean bond length of each trajectory. For each quantity, the change was calculated by subtracting the corresponding zero-field value from the laser-excited value. The results were obtained by analysing the first 12 electric-field oscillation cycles of the CW simulations and are presented as a function of laser electric-field amplitude for the selected vibrational modes described in Section 3.2. A static electric field is included as a reference. Error bars represent the standard deviation of the changes across ten independent trajectory pairs.

The thermal effects discussed in the previous section demonstrate that infrared laser excitation can deposit substantial energy into aqueous CO_2_–amine systems through strongly mode-selective vibrational heating. However, thermal energy deposition represents only one mechanism by which infrared laser excitation may influence amine regeneration. Resonant excitation of selected vibrational modes may also directly perturb the chemical bonds responsible for retaining the CO_2_ molecules, producing structural and energetic responses that are not simply reflected by the overall temperature increase. To investigate these effects, the nonequilibrium Ehrenfest electron–nuclear dynamics simulations initiated at 0 K were analysed over the first 12 electric-field oscillation cycles of the CW excitation. This early-time analysis reduces the contribution of cumulative laser-induced heating and improves sensitivity to mode-selective structural and energetic changes. The regeneration-relevant bond lengths, bond-length distributions, probabilities of elongated-bond configurations, and bond energies were analysed relative to the corresponding zero-field trajectories to assess whether mode-selective infrared laser excitation can transiently destabilize the CO_2_ absorption products. The structural response is summarized in Figure 5.

To reduce the contribution of cumulative heating, the structural and energetic analyses were restricted to the first 12 cycles of the 30-cycle CW simulations. Starting from 0 K removes pre-existing thermal motion but does not eliminate the vibrational kinetic energy deposited by the infrared laser itself. Accordingly, the observed bond perturbations cannot be considered completely independent of localized vibrational heating. Here, the term “nonthermal” refers to mode-selective structural and energetic responses that cannot be explained by the overall kinetic-temperature increase alone, rather than to a strictly thermal-free mechanism.

In Figure 5, panels (a–c) show the laser-induced change in the mean length of the regeneration-relevant bond, corresponding to the C_CO_2__–N_MEA_ bond for the zwitterion–water and carbamate–water systems and the C_CO_2__–O_OH_ bond for the bicarbonate–water system. Panels (d–f) show the corresponding change in the root-mean-square (RMS) bond-length fluctuation. For each trajectory, the mean bond length and RMS fluctuation were calculated from the sampled bond lengths, and the corresponding zero-field values were subtracted to obtain the laser-induced changes. Positive changes indicate an increase in the mean bond length or greater bond-length fluctuations, respectively. By monitoring the same regeneration-relevant bond while selectively exciting different vibrational modes, Figure 5 identifies which modes produce the strongest structural perturbations relevant to CO_2_ release.

For the zwitterion–water system (Figure 5a,d), excitation of the C_CO_2__–O_CO_2__–C_CO_2__ bending mode produces the largest increase in the mean C_CO_2__–N_MEA_ bond length, closely followed by direct excitation of the C_CO_2__–N_MEA_ stretching mode. The symmetric C_CO_2__–O_CO_2__ stretching mode produces a smaller but still appreciable increase, whereas the asymmetric C_CO_2__–O_CO_2__ stretching mode produces only a minor change and the H_MEA_–N_MEA_ stretching mode decreases the mean bond length. The RMS bond-length fluctuation shows a similar strong mode dependence, with the C_CO_2__–O_CO_2__–C_CO_2__ bending and C_CO_2__–N_MEA_ stretching modes producing the greatest increases. The symmetric C_CO_2__–O_CO_2__ stretching mode also enhances bond-length fluctuations, while the asymmetric C_CO_2__–O_CO_2__ and H_MEA_–N_MEA_ stretching modes produce much smaller changes. These results show that excitation of the CO_2_-centred bending and C_CO_2__–N_MEA_ stretching modes is particularly effective at increasing both the mean C_CO_2__–N_MEA_ bond length and its fluctuations.

For the carbamate–water system (Figure 5b,e), direct excitation of the C_CO_2__–N_MEA_ stretching mode produces the strongest structural response of the regeneration-relevant C_CO_2__–N_MEA_ bond. Excitation of the asymmetric C_CO_2__–O_CO_2__ stretching mode produces the second-largest increase in the mean bond length, followed by the C_CO_2__–O_CO_2__–C_CO_2__ bending mode, while the symmetric C_CO_2__–O_CO_2__ stretching mode produces only a minor increase and the H_MEA_–N_MEA_ stretching mode decreases the mean bond length. The RMS bond-length fluctuation shows an even stronger mode dependence: direct excitation of the C_CO_2__–N_MEA_ stretch produces the largest increase, followed by the asymmetric C_CO_2__–O_CO_2__ stretch, whereas the remaining modes produce considerably smaller changes. Thus, for the carbamate system, direct excitation of the C_CO_2__–N_MEA_ stretching mode produces the largest increases in both the mean C_CO_2__–N_MEA_ bond length and its fluctuations.

For the bicarbonate–water system (Figure 5c,f), excitation of the symmetric C_CO_2__–O_CO_2__ stretching mode produces the strongest overall structural response of the regeneration-relevant C_CO_2__–O_OH_ bond. The C_CO_2__–O_CO_2__–C_CO_2__ bending mode also produces a substantial increase in the mean bond length, while direct excitation of the C_CO_2__–O_OH_ stretch and the asymmetric C_CO_2__–O_CO_2__ stretch give smaller responses. In contrast, excitation of the O_OH_–H_OH_ stretching mode produces a minor decrease in the mean bond length. The RMS bond-length fluctuation shows a clearer separation between the modes, with the symmetric C_CO_2__–O_CO_2__ stretch producing by far the largest increase. The remaining CO_2_-centred modes also enhance bond-length fluctuations, but to a considerably smaller extent. Thus, for the bicarbonate system, excitation of the symmetric C_CO_2__–O_CO_2__ stretching mode is particularly effective at increasing both the mean C_CO_2__–O_OH_ bond length and its fluctuations.

**Figure 6:**
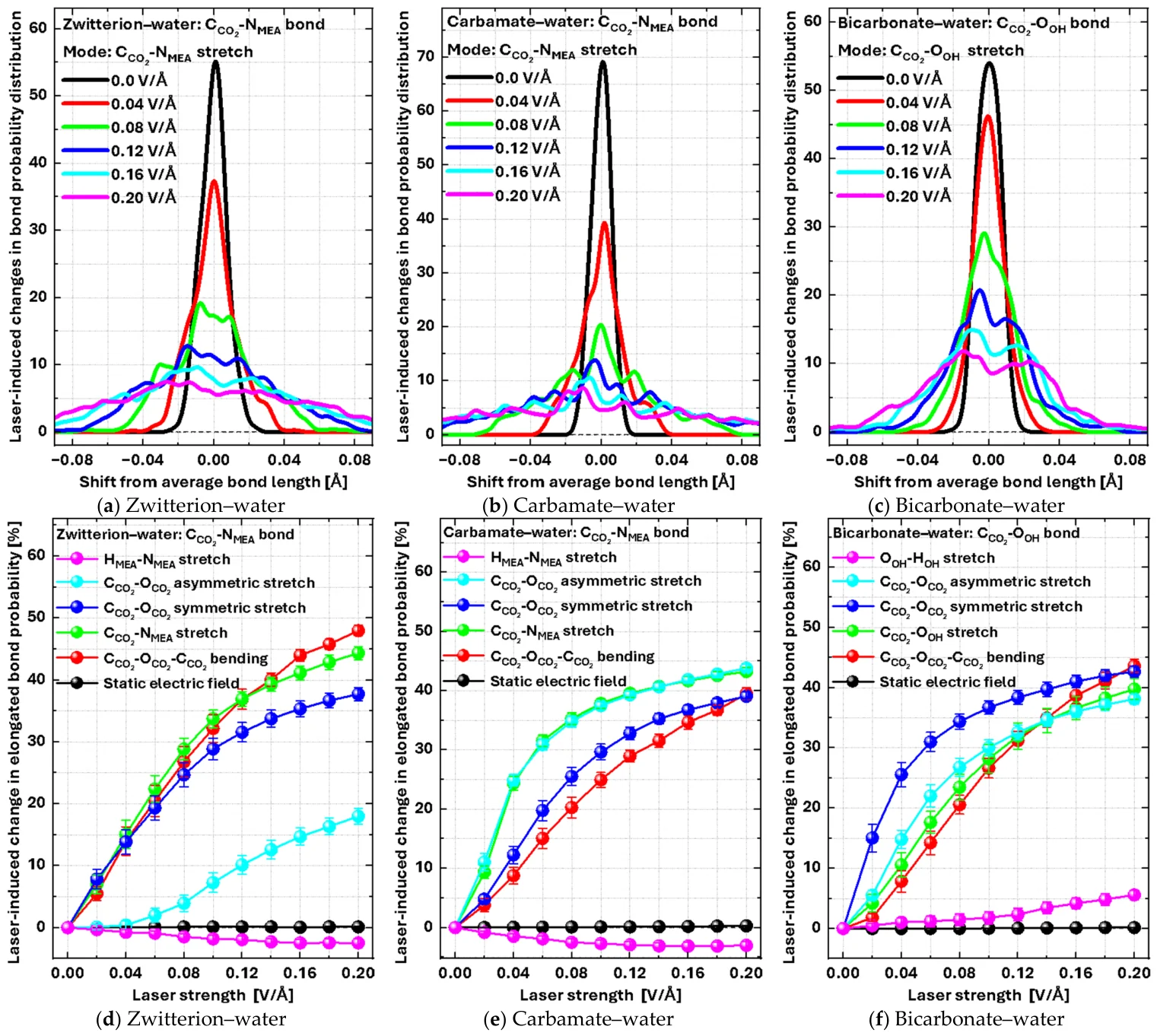
Infrared laser-induced changes in the bond-length distributions and elongated-bond probabilities of the regeneration-relevant bonds under CW excitation initiated at 0 K. Panels (**a**–**c**) show normalized distributions of instantaneous bond-length deviations from the corresponding mean bond length for the zwitterion–water, carbamate–water, and bicarbonate–water systems, respectively, at selected electric-field amplitudes from 0.00 to 0.20 V/Å. The illustrated excitation modes are the C_CO_2__–N_MEA_ stretch for the zwitterion and carbamate systems and the C_CO_2__–O_OH_ stretch for the bicarbonate system. Panels (**d**–**f**) show the laser-induced change in elongated-bond probability, calculated by subtracting the corresponding zero-field probability from the laser-excited probability. An elongated bond is defined as one whose length exceeds the mean bond length of its corresponding zero-field trajectory by 1.5 standard deviations. Probability changes are expressed as differences in percentage points. A static electric field is included as a reference in panels (**d**–**f**). The results were obtained by analysing the first 12 electric-field oscillation cycles of the CW simulations. Error bars represent the standard deviations of the probability changes across ten independent trajectory pairs. Absolute elongated-bond probabilities and bond-length distribution widths are reported in Tables S1–S6 of the Supporting Information.

Although the changes in mean bond length and RMS bond-length fluctuation shown in Figure 5 characterize the structural response, they do not directly quantify how frequently the regeneration-relevant bonds reach elongated configurations. To examine this, an elongated bond was defined as one whose length exceeds the corresponding zero-field mean by 1.5 standard deviations (r > $\bar{r_{0}}$ + 1.5σ_0_). This threshold was determined separately for each zero-field trajectory and then applied to both that trajectory and its matching laser-excited trajectory. The elongated-bond probability was calculated as the percentage of sampled bond lengths exceeding the threshold, and the laser-induced change was obtained by subtracting the zero-field probability from the laser-excited probability. Figure 6 presents representative bond-length distributions and the resulting changes in elongated-bond probability, while Tables S1–S6 in the Supporting Information report the absolute probabilities and bond-length distribution widths.

The representative bond-length distributions in Figure 6a–c show that increasing the infrared laser field amplitude generally broadens the range of configurations sampled by the regeneration-relevant bonds. This behaviour is evident for excitation of the C_CO_2__–N_MEA_ stretch in both the zwitterion and carbamate systems and the C_CO_2__–O_OH_ stretch in the bicarbonate system. In each case, the initially narrow distribution becomes lower and wider as the field amplitude increases, indicating greater bond-length fluctuations. However, broadening includes excursions toward both shorter and longer bond lengths and does not, by itself, establish bond weakening. The positive-deviation tail represents transiently elongated bond configurations. The lower panels of Figure 6 therefore quantify how the probability of sampling these configurations changes with laser field amplitude for each investigated vibrational mode.

The lower panels of Figure 6 show that the laser-induced change in elongated-bond probability depends strongly on the excited vibrational mode and the CO_2_ absorption product. For the zwitterion–water system (Figure 6d), the C_CO_2__–N_MEA_ stretching and symmetric C_CO_2__–O_CO_2__ stretching modes produce substantial increases at low-to-intermediate field amplitudes, while the C_CO_2__–O_CO_2__–C_CO_2__ bending mode produces the largest increase at higher amplitudes, reaching approximately 47.3 percentage points at 0.20 V/Å. For the carbamate–water system (Figure 6e), the C_CO_2__–N_MEA_ and asymmetric C_CO_2__–O_CO_2__ stretching modes produce the largest increases, with the asymmetric stretch reaching approximately 43.8 percentage points at 0.20 V/Å. For the bicarbonate–water system (Figure 6f), the symmetric C_CO_2__–O_CO_2__ stretch produces the largest increase over most of the investigated field range, but the C_CO_2__–O_CO_2__–C_CO_2__ bending mode gives a slightly larger increase at 0.20 V/Å, reaching approximately 43.6 percentage points. Excitation of the H_MEA_–N_MEA_ stretch decreases the elongated-bond probability in the zwitterion and carbamate systems, whereas the H_OH_–O_OH_ stretch produces a smaller positive change in the bicarbonate system. The static electric field produces only minor changes. These results show that excitation of modes other than the monitored bond stretch can substantially increase the frequency of transient elongated-bond configurations.

**Figure 7:**
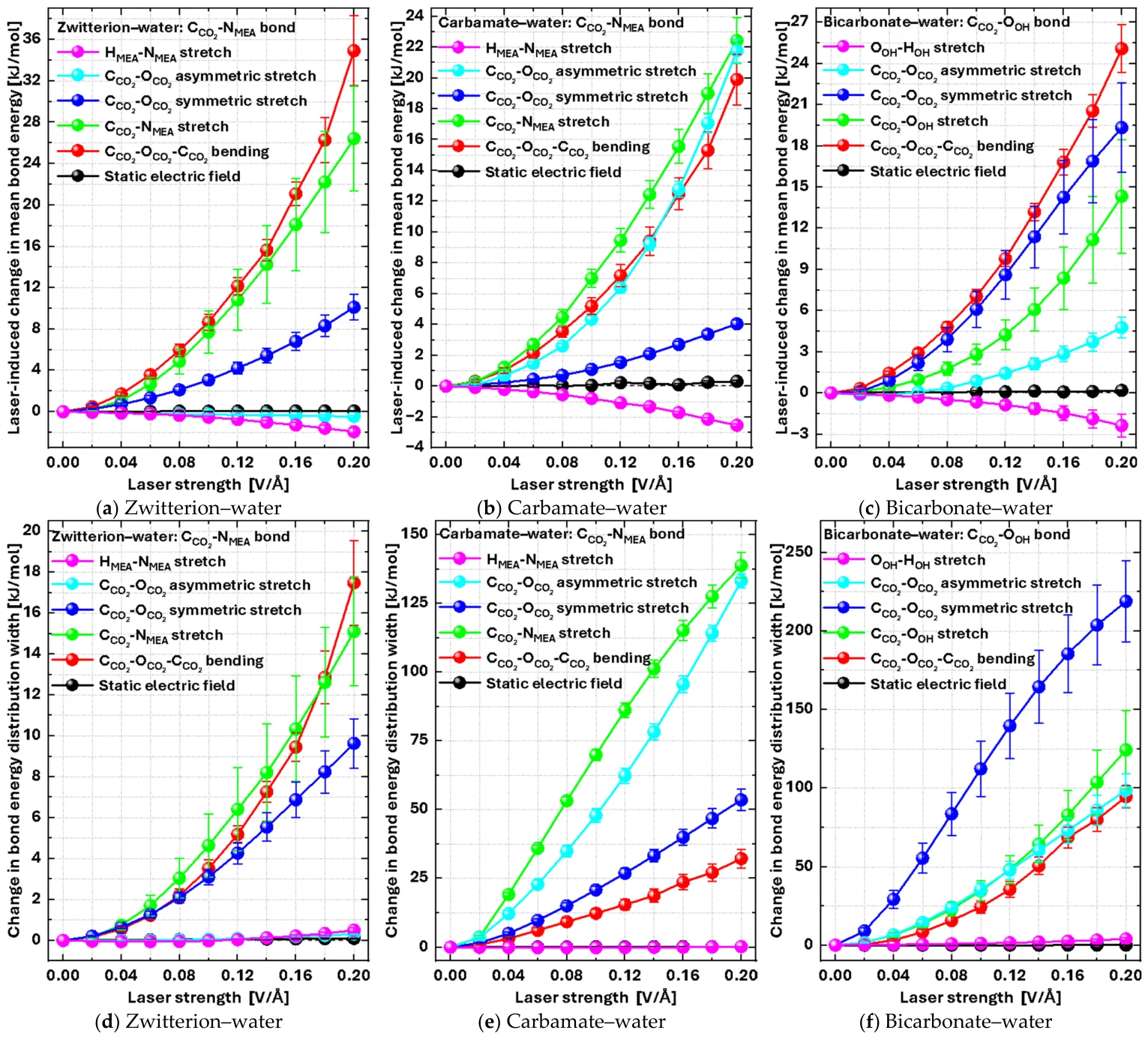
Nonthermal energetic response of the regeneration-relevant bonds in the aqueous zwitterion–water (**a**,**d**), carbamate–water (**b**,**e**), and bicarbonate–water (**c**,**f**) systems under CW infrared laser excitation initiated at 0 K. Panels (**a**–**c**) show the laser-induced change in the mean bond energy of the C_CO_2__–N_MEA_ bond for the zwitterion and carbamate systems and the C_CO_2__–O_OH_ bond for the bicarbonate system. A positive change indicates bond weakening. Panels (**d**–**f**) show the corresponding change in bond-energy distribution width, defined as the standard deviation of the sampled bond energies. Both changes are expressed in kJ/mol and calculated by subtracting the corresponding zero-field values from the laser-excited values. The results were obtained by analysing the first 12 electric-field oscillation cycles of the CW simulations and are presented as a function of laser electric-field amplitude for the selected vibrational modes described in Section 3.2. A static electric field is included as a reference. Error bars represent the standard deviations of the changes across ten independent trajectory pairs.

To complement the structural analysis in Figure 5 and Figure 6, Figure 7 examines the energetic response of the same regeneration-relevant bonds. The bond-energy analysis provides a measure of how infrared laser excitation perturbs the C_CO_2__–N_MEA_ bond in the zwitterion and carbamate systems and the C_CO_2__–O_OH_ bond in the bicarbonate system. For each trajectory, the mean bond energy and the standard deviation of its sampled values were calculated from the DFTB+ pairwise electronic bond-energy contributions. The corresponding zero-field values were then subtracted to obtain the laser-induced changes, expressed in kJ/mol. Panels (a–c) show the change in mean bond energy, where a positive value indicates a less negative bond-energy contribution and hence bond weakening. Panels (d–f) show the change in bond-energy distribution width, which measures the change in temporal energy fluctuations. Together, these quantities provide an energetic counterpart to the structural measures discussed above.

For the zwitterion–water system (Figure 7a,d), excitation of the C_CO_2__–O_CO_2__–C_CO_2__ bending mode produces the largest increase in the mean energy of the C_CO_2__–N_MEA_ bond, reaching approximately 35 kJ/mol at 0.20 V/Å. Direct excitation of the C_CO_2__–N_MEA_ stretching mode produces the second-largest increase, reaching approximately 26 kJ/mol, followed by the symmetric C_CO_2__–O_CO_2__ stretching mode at approximately 10 kJ/mol. The asymmetric C_CO_2__–O_CO_2__ stretch produces little change, whereas the H_MEA_–N_MEA_ stretch slightly decreases the mean bond energy. The bond-energy distribution width also increases strongly under excitation of the bending and C_CO_2__–N_MEA_ stretching modes, reaching approximately 18 and 15 kJ/mol, respectively, at the highest field amplitude. The symmetric C_CO_2__–O_CO_2__ stretch produces a smaller increase, while the remaining modes have little effect. These results indicate that excitation of the bending mode produces the greatest average energetic weakening of the C_CO_2__–N_MEA_ bond, while both the bending and C_CO_2__–N_MEA_ stretching modes substantially enhance its energetic fluctuations.

For the carbamate–water system (Figure 7b,e), direct excitation of the C_CO_2__–N_MEA_ stretching mode produces the largest increase in the mean C_CO_2__–N_MEA_ bond energy, reaching approximately 23 kJ/mol at 0.20 V/Å. Excitation of the C_CO_2__–O_CO_2__–C_CO_2__ bending and asymmetric C_CO_2__–O_CO_2__ stretching modes also produces substantial increases, reaching approximately 20 and 17 kJ/mol, respectively. The symmetric C_CO_2__–O_CO_2__ stretch produces a smaller increase, whereas the H_MEA_–N_MEA_ stretch decreases the mean bond energy. The bond-energy distribution width shows a different ordering: the C_CO_2__–N_MEA_ and asymmetric C_CO_2__–O_CO_2__ stretching modes produce the largest increases, reaching approximately 140 and 135 kJ/mol, respectively, at the highest field amplitude. The symmetric C_CO_2__–O_CO_2__ stretch produces a smaller increase, followed by the bending mode. Thus, direct excitation of the C_CO_2__–N_MEA_ stretching mode produces the largest increases in both the mean bond energy and its fluctuations, while excitation of the asymmetric C_CO_2__–O_CO_2__ stretch also strongly enhances energetic fluctuations.

For the bicarbonate–water system (Figure 7c,f), excitation of the C_CO_2__–O_CO_2__–C_CO_2__ bending mode produces the largest increase in the mean energy of the regeneration-relevant C_CO_2__–O_OH_ bond, reaching approximately 25 kJ/mol at 0.20 V/Å. The symmetric C_CO_2__–O_CO_2__ stretching mode produces the second-largest increase, reaching approximately 19 kJ/mol, followed by direct excitation of the C_CO_2__–O_OH_ stretch at approximately 14 kJ/mol. The asymmetric C_CO_2__–O_CO_2__ stretch produces a smaller increase, whereas the H_OH_–O_OH_ stretch decreases the mean bond energy. The bond-energy distribution width shows a different ordering: the symmetric C_CO_2__–O_CO_2__ stretch produces by far the largest increase, reaching approximately 220 kJ/mol at the highest field amplitude. Direct excitation of the C_CO_2__–O_OH_ stretch produces the second-largest increase, reaching approximately 125 kJ/mol, followed by the asymmetric C_CO_2__–O_CO_2__ stretching and bending modes, which both produce increases of approximately 100 kJ/mol. Thus, the bending mode produces the greatest average energetic weakening of the C_CO_2__–O_OH_ bond, whereas the symmetric C_CO_2__–O_CO_2__ stretch produces the largest increase in its energetic fluctuations.

Figure 7 demonstrates that the energetic response of the regeneration-relevant bonds is strongly mode selective and differs substantially among the three CO_2_ absorption products. For the zwitterion, the C_CO_2__–O_CO_2__–C_CO_2__ bending mode produces the largest increase in the mean C_CO_2__–N_MEA_ bond energy, while both the bending and C_CO_2__–N_MEA_ stretching modes strongly enhance its energetic fluctuations. For the carbamate, direct excitation of the C_CO_2__–N_MEA_ stretching mode produces the largest increases in both energetic descriptors, although the asymmetric C_CO_2__–O_CO_2__ stretch also strongly enhances the bond-energy fluctuations. The bicarbonate system exhibits a different response: the C_CO_2__–O_CO_2__–C_CO_2__ bending mode produces the largest increase in the mean C_CO_2__–O_OH_ bond energy, whereas the symmetric C_CO_2__–O_CO_2__ stretching mode produces the greatest increase in its energy-distribution width. These differences show that energetic perturbations of regeneration-relevant bonds do not require direct excitation of those bonds and may also arise through coupling to other vibrational modes. Together with the structural responses shown in Figure 5 and Figure 6, these results demonstrate that mode-selective infrared laser excitation can enhance both geometric and energetic fluctuations of bonds involved in CO_2_ retention.

In summary, the nonthermal effects presented in Figure 5, Figure 6 and Figure 7 demonstrate that infrared laser excitation produces strongly mode-selective structural and energetic perturbations of the CO_2_ absorption products. Selected vibrational modes increase the mean lengths and energies of the regeneration-relevant C_CO_2__–N_MEA_ and C_CO_2__–O_OH_ bonds, broaden their bond-length and bond-energy distributions, and increase the probability of sampling transient elongated-bond configurations. Importantly, the excitation mode producing the strongest response differs among the zwitterion, carbamate, and bicarbonate systems and is not always the mode directly associated with the monitored bond. These findings are consistent with vibrational coupling and redistribution of absorbed energy among different molecular modes. In contrast to the stabilizing influence of static electric fields identified in Part I [19], resonant infrared laser excitation promotes transient access to structurally distorted and energetically weakened configurations of the CO_2_ absorption products. Combined with the mode-selective heating discussed in Section 2.1, these results indicate that infrared laser excitation can influence amine regeneration through both thermal energy deposition and mode-dependent bond perturbations that are not explained by the overall kinetic-temperature increase alone.

### 2.3. Mode-Selective Infrared Laser Excitation and Its Implications for Amine Regeneration

The combined thermal and nonthermal results demonstrate that the response of CO_2_ absorption products to infrared laser excitation cannot be described by the overall temperature increase alone. Although resonant excitation increases the overall kinetic energy, the subsequent distribution of the absorbed energy depends strongly on both the excited vibrational mode and the identity of the CO_2_ absorption product. Consequently, the vibrational mode that produces the greatest temperature increase is not necessarily the one that produces the strongest structural or energetic perturbations of the regeneration-relevant bond. Evaluating the potential of infrared laser-assisted amine regeneration therefore requires consideration of both thermal energy deposition and mode-selective bond perturbations that are not explained by the overall kinetic-temperature increase alone.

This distinction arises because the thermal and nonthermal responses reflect different aspects of vibrational energy absorption and redistribution. Laser-induced heating depends not only on how efficiently a particular mode absorbs energy, but also on how that energy is partitioned between the CO_2_–amine species and the surrounding water. As shown in Figure 4, this partitioning is strongly mode and system dependent: some excitation modes preferentially heat the CO_2_–amine species, whereas others transfer comparable or greater vibrational energy to the surrounding water. This distinction is particularly important for regeneration because preferential heating of the CO_2_–amine species concentrates the supplied energy within the molecular species directly involved in CO_2_ release, whereas preferential solvent heating contributes more strongly to bulk thermalization. At the same time, the nonthermal response depends on how efficiently vibrational excitation is transferred into structural and energetic fluctuations of the regeneration-relevant bond. Consequently, the mode producing the greatest total-system heating is not necessarily the mode that preferentially heats the CO_2_–amine species or produces the strongest structural and energetic perturbations of the relevant bonds.

The C_CO_2__–O_CO_2__–C_CO_2__ bending mode illustrates this distinction particularly clearly: it consistently provides the strongest overall heating across all three systems, yet in the carbamate–water and bicarbonate–water systems it preferentially deposits more vibrational energy into the surrounding water. At the same time, excitation of this mode produces substantial structural and energetic perturbations of the regeneration-relevant bonds, demonstrating that preferential solvent heating does not preclude a strong response of the CO_2_ absorption products. This behaviour is consistent with the established concept of mode-selective vibrational energy redistribution, whereby energy initially deposited into one vibrational mode can be redistributed through coupling with other molecular motions and thereby influence chemically relevant degrees of freedom that are not directly excited [29,30,31,32,33]. Thus, total heating efficiency, preferential energy deposition into the CO_2_–amine species relative to water, and mode-selective bond perturbations represent three distinct but complementary aspects of infrared excitation.

The relative importance of these three effects differs substantially among the zwitterion, carbamate, and bicarbonate systems, indicating that no single vibrational mode maximizes all three responses across the investigated systems. For the zwitterion, the symmetric C_CO_2__–O_CO_2__ stretch produces the strongest preferential heating of the CO_2_–amine species, whereas the C_CO_2__–O_CO_2__–C_CO_2__ bending mode provides the greatest total-system heating and produces substantial structural and energetic perturbations of the C_CO_2__–N_MEA_ bond. Direct excitation of the C_CO_2__–N_MEA_ stretch also preferentially heats the zwitterion and strongly enhances C_CO_2__–N_MEA_ bond fluctuations. For the carbamate, the C_CO_2__–N_MEA_ stretch preferentially deposits vibrational energy into the carbamate-containing species and produces the largest increases in the mean length and energy of the C_CO_2__–N_MEA_ bond, as well as its structural and energetic fluctuations. The asymmetric C_CO_2__–O_CO_2__ stretch also preferentially heats the carbamate species and produces substantial bond perturbations. For the bicarbonate, the C_CO_2__–O_OH_ stretch produces the strongest preferential heating of the bicarbonate-containing species, whereas the symmetric C_CO_2__–O_CO_2__ stretch produces the largest increases in the mean C_CO_2__–O_OH_ bond length and its structural and energetic fluctuations. The C_CO_2__–O_CO_2__–C_CO_2__ bending mode provides the greatest total-system heating and produces the largest increase in the mean C_CO_2__–O_OH_ bond energy. These results indicate that selecting an excitation frequency for infrared-assisted regeneration requires consideration of overall heating, preferential energy deposition into the CO_2_–amine species, and structural and energetic perturbations of the bond involved in CO_2_ retention.

The present study therefore suggests that selecting an excitation frequency for infrared laser-assisted amine regeneration is inherently a multi-objective problem. A mode that maximizes total-system heating may preferentially deposit a substantial fraction of the absorbed energy into the surrounding water, while a mode that selectively heats the CO_2_–amine species may not necessarily produce the strongest structural and energetic perturbations of the regeneration-relevant bond. Conversely, a mode capable of generating pronounced bond-length or bond-energy fluctuations may contribute only moderately to overall heating. The selection of an excitation mode should therefore consider three characteristics: efficient absorption of infrared laser energy, preferential deposition of that energy into the CO_2_–amine species rather than the solvent, and structural and energetic perturbations of the bonds involved in CO_2_ retention. The relative importance of these contributions may differ among absorption products and operating conditions, and establishing their optimal balance lies beyond the scope of the present work. Nevertheless, the descriptors developed here provide a molecular-level framework for evaluating excitation frequencies and laser excitation protocols for infrared-assisted amine regeneration.

Finally, the present study reveals a fundamentally different response from that observed under static electric fields in Part I. Static electric fields were found to stabilize the CO_2_ absorption products and increase the energetic requirements for regeneration, whereas infrared laser excitation drives the systems away from equilibrium through mode-selective vibrational excitation and energy redistribution. Rather than imposing a persistent stabilizing perturbation, the oscillating field can redistribute absorbed energy among molecular degrees of freedom and promote transient bond elongation and energetic changes associated with weakening of the regeneration-relevant bonds. Infrared laser-assisted regeneration should therefore be viewed not simply as an alternative means of supplying thermal energy, but as a mode-selective process in which the magnitude, location, and redistribution of the absorbed energy are all important. Together, these findings identify vibrational mode selectivity, preferential energy deposition, and mode-dependent bond perturbations as important molecular-level considerations for developing infrared laser-assisted amine regeneration strategies.

It is important to distinguish the molecular-level advantages identified here from the overall energy efficiency of an infrared laser-assisted regeneration process. Frequency-selective infrared excitation can direct energy toward CO_2_–amine vibrational modes and bonds involved in CO_2_ release, potentially reducing unnecessary bulk heating. However, the present simulations do not include the energy required to operate the laser and therefore do not demonstrate a net process-level energy saving. This will require experimental validation and full process-level energy analysis. Experimental validation will also be important given the approximate nature of TD-DFTB. The present results should therefore be interpreted mainly in terms of molecular mechanisms and relative mode-dependent trends rather than quantitatively exact heating rates. Frequency-selective photothermal or calorimetric measurements could test the predicted mode-dependent differences in energy deposition and provide experimental validation of the simulated heating trends.

## 3. Materials and Methods

### 3.1. Benchmarking of Semiempirical Methods

Semiempirical computational chemistry calculations were performed using version 25.1 of the DFTB+ software package [26]. All calculations were carried out on the Eddie high-performance computing cluster at the University of Edinburgh. A systematic benchmarking study was undertaken to evaluate the predictive performance of selected semiempirical computational chemistry methods implemented in DFTB+, including the MIO, 3OB, OB2, and 3OB-FREQ tight-binding parameterizations, together with the GFN1-xTB and GFN2-xTB extended tight-binding methods. The primary objective was to identify the most reliable semiempirical approach for the subsequent time-dependent Ehrenfest electron–nuclear dynamics simulations under external infrared laser excitation.

**Figure 8:**
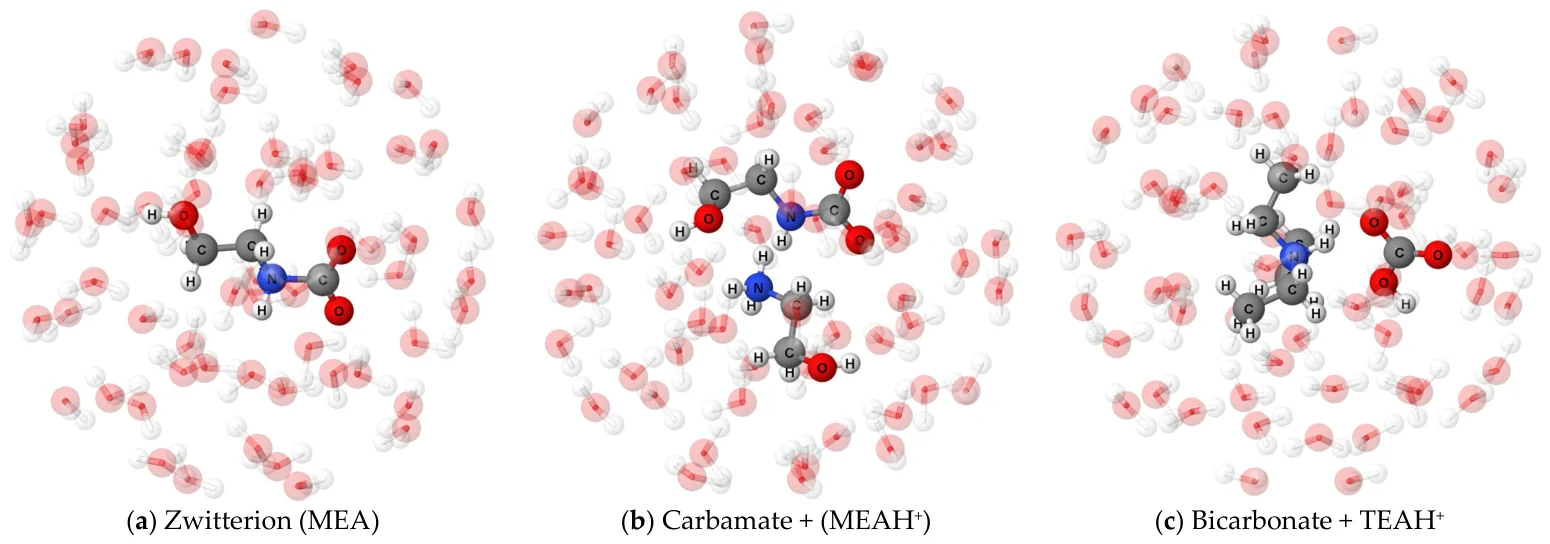

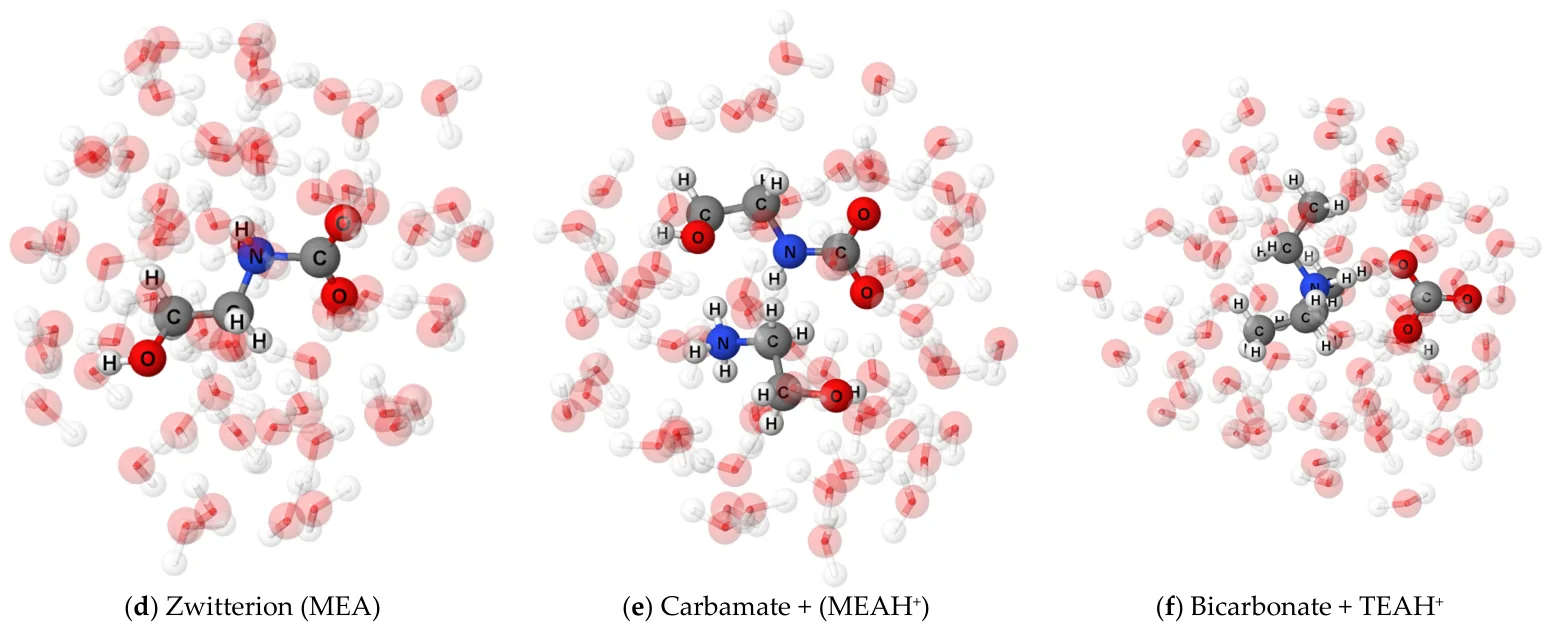
Initial (**a**–**c**) and optimized (**d**–**f**) geometries of the aqueous CO_2_–amine absorption products considered for the MEA and TEA systems: zwitterion (**a**,**d**), carbamate with protonated MEA (MEAH^+^) (**b**,**e**), and bicarbonate with protonated TEA (TEAH^+^) (**c**,**f**). The optimized structures were obtained using the 3OB-FREQ parameter set. Explicit water molecules are shown as semi-transparent to improve visualization of the CO_2_–amine absorption products.

For each semiempirical method, full geometry optimizations were performed for the aqueous CO_2_–amine absorption products to identify their minimum-energy structures. The resulting optimized geometries were then used to evaluate key structural and vibrational properties, including selected bond lengths, bond angles, and harmonic vibrational frequencies. These quantities were chosen as the principal benchmarking metrics because they directly determine the equilibrium molecular structures and influence the response of the systems to external infrared laser excitation. For MEA, the zwitterion, carbamate, and protonated MEA (MEAH^+^) species were considered, while for TEA, bicarbonate and protonated TEA (TEAH^+^) were examined. In all cases, explicit solvation was represented by 60 water molecules surrounding the CO_2_–amine absorption products. The initial and optimized geometries are shown in Figure 8 for the zwitterion (Figure 8a,d), carbamate with protonated MEA (Figure 8b,e), and bicarbonate with protonated TEA (Figure 8c,f). Initial configurations were generated using PACKMOL version 20.14.3 [34], and the optimized structures presented in Figure 8 were obtained using the 3OB-FREQ parameter set. All calculations were performed for isolated molecular clusters without periodic boundary conditions.

**Figure 9:**
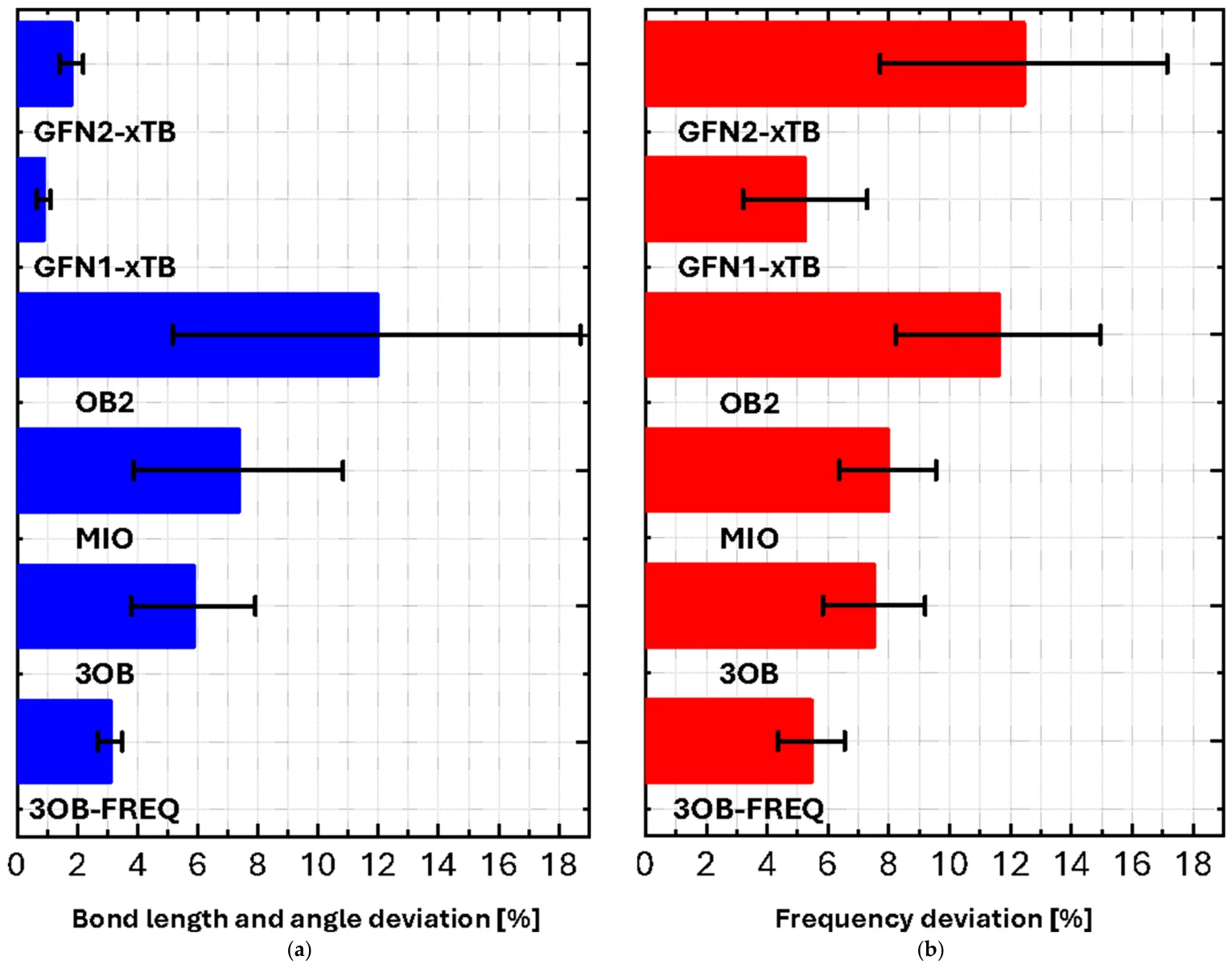
Mean absolute percentage deviations of the MIO, 3OB, OB2, 3OB-FREQ, GFN1-xTB, and GFN2-xTB methods relative to the reference DFT results. Left figure (**a**) shows the average deviations in the selected bond lengths and bond angles, while right figure (**b**) shows the average deviations in the selected harmonic vibrational frequencies. Error bars represent the variation in the percentage deviations across the evaluated structural and vibrational properties.

The benchmarking results obtained in this study are summarized in Table 1 and Table 2 and Figure 9. Table 1 reports selected structural parameters, including representative bond lengths and bond angles for water and the CO_2_–amine absorption products, while Table 2 presents the corresponding harmonic vibrational frequencies for characteristic stretching and bending modes. The semiempirical results are compared directly with reference DFT calculations from Part I of this series [19] to assess the accuracy of each method. To provide a quantitative comparison, Figure 9 presents the mean absolute percentage deviations relative to the DFT reference, with left figure (a) showing the average deviations in bond lengths and bond angles and right figure (b) showing the average deviations in vibrational frequencies. Together, these results provide a systematic assessment of the performance of the MIO, 3OB, OB2, 3OB-FREQ, GFN1-xTB, and GFN2-xTB methods for describing the structural and vibrational properties of the aqueous CO_2_–amine systems considered in this work.

For the structural properties, clear differences in performance are observed among the tested methods. As shown in Table 1 and Figure 9a, GFN1-xTB provides the most accurate overall description of the selected bond lengths and bond angles, exhibiting the smallest mean deviations relative to the DFT reference. This is particularly evident for the MEA zwitterion, for which GFN1-xTB closely reproduces both the C_CO_2__–N_MEA_ bond length and the C_CO_2__–O_CO_2__–C_CO_2__ bond angle, whereas several other methods, especially OB2, show substantially larger deviations. GFN2-xTB also yields good structural agreement, although its deviations are generally larger than those of GFN1-xTB. This performance is consistent with the design of the GFN-xTB family, which was parameterized to provide reliable molecular geometries and noncovalent interactions across a broad range of chemical systems [27,28]. Among the DFTB-based parameterizations, 3OB-FREQ provides the most balanced structural performance, improving upon 3OB, MIO, and OB2, particularly for the zwitterionic species, where an accurate description of the C_CO_2__–N_MEA_ bond and CO_2_ geometry is essential. By contrast, OB2 exhibits relatively large errors for several structural parameters, including overestimated bond lengths and distorted bond angles, indicating reduced transferability to the CO_2_–amine systems considered here.

The vibrational-frequency benchmarking results reveal a similar, although not identical, trend. As shown in Table 2 and Figure 9b, GFN1-xTB again exhibits the lowest overall deviation from the DFT reference results, confirming its strong performance for both structural and vibrational properties. However, 3OB-FREQ performs comparably well for many of the modes considered, particularly the CO_2_ stretching and bending vibrations, consistent with its parameterization for improved vibrational properties [35]. The standard 3OB and MIO parameter sets show moderate deviations, whereas OB2 and GFN2-xTB exhibit larger discrepancies for several selected vibrational modes. Although GFN2-xTB generally provides accurate structural properties, its vibrational frequencies are less consistent, especially for some higher-frequency stretching modes, indicating that improvements in electrostatics and dispersion do not necessarily yield uniformly better vibrational predictions for all systems considered here [27,28].

These results highlight the system- and property-dependent performance of the evaluated semiempirical computational chemistry parameterizations and reinforce the importance of benchmarking both structural and vibrational properties before selecting a method for infrared laser-driven electron–nuclear dynamics simulations. The observed differences reflect the distinct parameterization strategies of the various methods, which place different emphasis on the accuracies of structural and vibrational properties [27,28,35]. Overall, the benchmarking results identify GFN1-xTB as the most accurate semiempirical method for the combined structural and vibrational properties considered in this study. Nevertheless, the subsequent time-dependent Ehrenfest electron–nuclear dynamics simulations were performed using the 3OB-FREQ parameterization because of a practical implementation constraint. The real-time Ehrenfest dynamics functionality employed in DFTB+ is currently available for DFTB-based Hamiltonians, whereas GFN1-xTB is not fully supported within the corresponding workflow [26]. Consequently, 3OB-FREQ provides the most suitable practical compromise, offering the best overall agreement with the DFT reference results among the tested DFTB parameterizations while remaining fully compatible with the nonequilibrium electron–nuclear dynamics simulations required for infrared laser excitation.

### 3.2. Nonequilibrium Ehrenfest Electron–Nuclear Dynamics Simulations

All subsequent simulations were performed using the DFTB+ code with the 3OB-FREQ parameter set. To generate equilibrated configurations for the nonequilibrium time-dependent Ehrenfest electron–nuclear dynamics simulations, molecular dynamics equilibration was carried out for three representative systems: a zwitterion solvated in water, a carbamate with protonated MEA solvated in water, and a bicarbonate with protonated TEA solvated in water. Each system contained one CO_2_–amine absorption product and 60 explicit water molecules. For clarity, these systems are hereafter referred to as zwitterion–water, carbamate–water, and bicarbonate–water, respectively.

**Figure 10:**
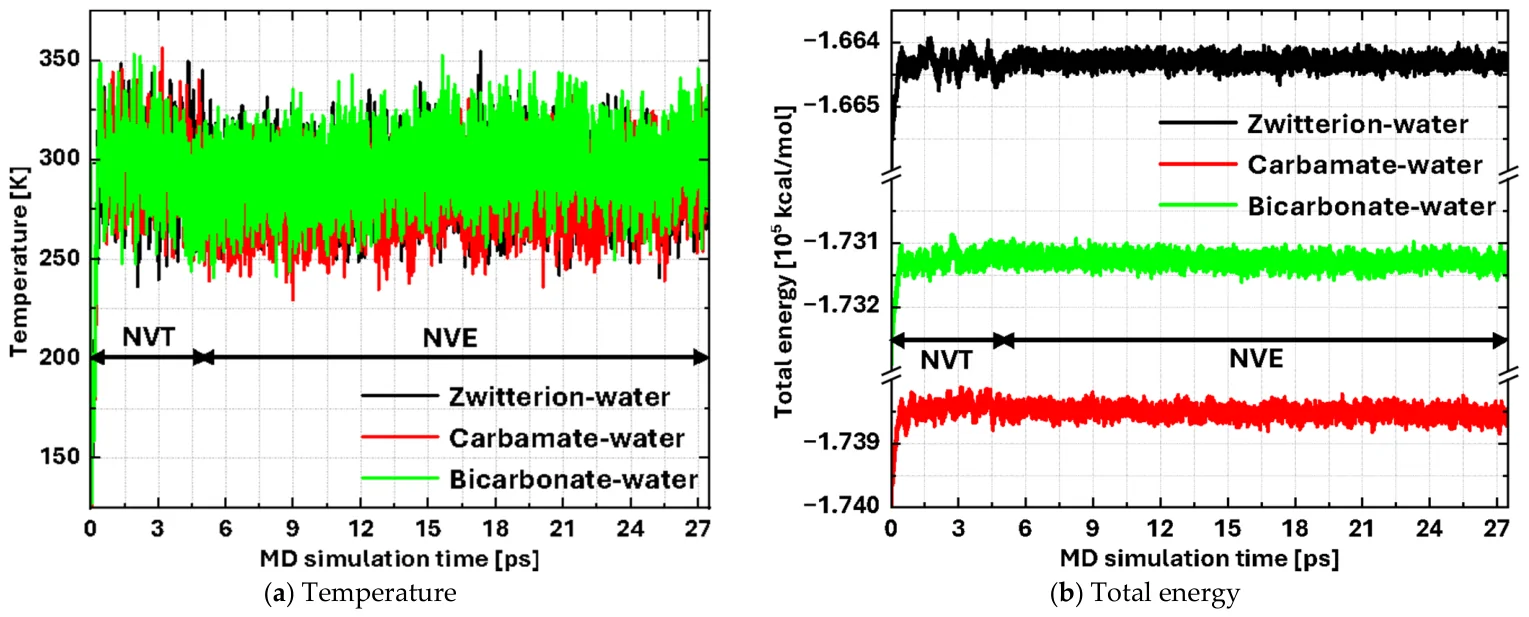
Temperature (**a**) and total-energy (**b**) profiles obtained during molecular dynamics equilibration of the zwitterion–water, carbamate–water, and bicarbonate–water systems. Each simulation comprised an initial 5 ps constant-number, constant-volume, constant-temperature (NVT) stage at 298.15 K, followed by a 22.5 ps constant-number, constant-volume, constant-energy (NVE) stage.

Molecular dynamics equilibration simulations were performed using a timestep of 0.05 fs. For each system, an initial constant-number, constant-volume, constant-temperature (NVT) simulation was carried out at 298.15 K for 5 ps to thermally equilibrate the system. This was followed by a 22.5 ps constant-number, constant-volume, constant-energy (NVE) simulation to assess energy conservation and generate equilibrated configurations. Ten independent configurations were then selected from the resulting NVE trajectories and separately geometry optimized using the 3OB-FREQ parameterization before being used as starting structures for the subsequent nonequilibrium Ehrenfest electron–nuclear dynamics simulations. Temperature control during the NVT stage was achieved using a Nosé–Hoover chain thermostat [36] with a coupling frequency of 500 cm^−1^, selected to provide relatively gentle thermalization. The resulting temperature and total-energy profiles for all three systems are presented in Figure 10 and confirm satisfactory equilibration before selection and geometry optimization of the configurations used for the subsequent simulations.

**Figure 11:**
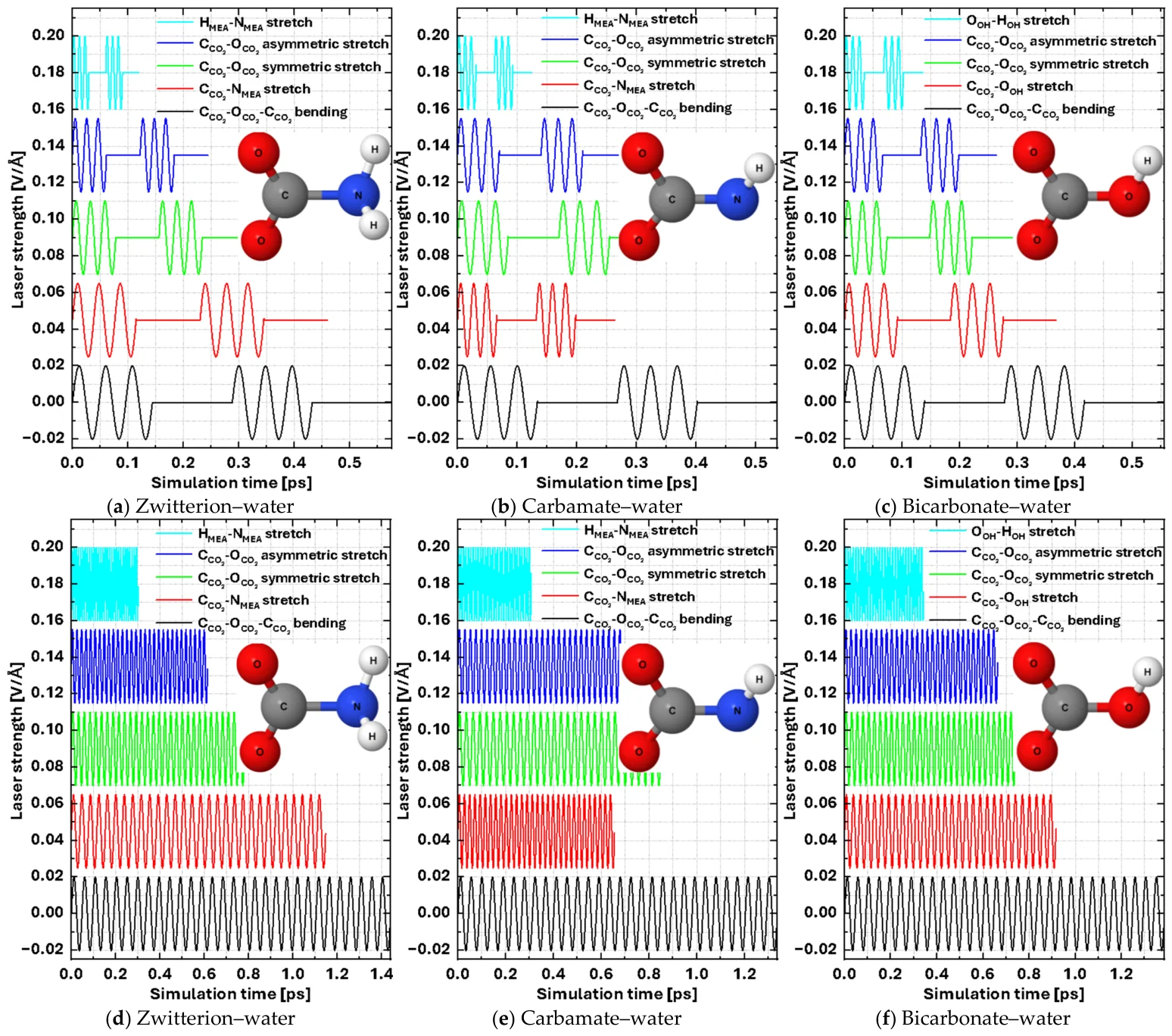
Infrared laser excitation signals applied during the nonequilibrium Ehrenfest electron–nuclear dynamics simulations of the zwitterion–water (**a**,**d**), carbamate–water (**b**,**e**), and bicarbonate–water (**c**,**f**) systems. Panels (**a**–**c**) show representative pulsed excitation signals for the vibrational modes investigated. Each pulse comprised three electric-field oscillation cycles followed by an equal field-free relaxation interval, with a total of 10 pulses applied during each simulation. Panels (**d**–**f**) show the corresponding continuous-wave (CW) excitation signals, comprising 30 continuous electric-field oscillation cycles. The excitation frequencies were selected to match characteristic stretching and bending modes of the CO_2_–amine absorption products.

Time-dependent Ehrenfest electron–nuclear dynamics simulations were performed under externally applied infrared laser excitation to investigate how different vibrational modes influence the nonequilibrium response of the CO_2_–amine systems. Two excitation protocols were considered: pulsed and continuous-wave (CW) infrared laser irradiation. These protocols represent distinct regimes of vibrational energy deposition and molecular response [29,37]. Pulsed excitation delivers energy intermittently, allowing partial relaxation between successive pulses and thereby promoting transient nonequilibrium dynamics. By contrast, CW excitation continuously drives the targeted vibrational modes and probes the cumulative effects of sustained resonant energy input [29,37]. Comparing the two protocols enables assessment of whether intermittent or continuous vibrational driving is more effective in heating the studied systems. In addition, comparing different excitation frequencies enables evaluation of vibrational mode selectivity, a concept long recognized as central to controlling molecular structure and reactivity through targeted vibrational excitation [30,31,32]. The laser signals applied to the zwitterion–water, carbamate–water, and bicarbonate–water systems under the pulsed and CW protocols are illustrated in Figure 11.

For each system, the optimized starting configuration was first oriented such that the regeneration-relevant bond was aligned with the x direction, corresponding to the C_CO_2__–N_MEA_ bond for the zwitterion–water and carbamate–water systems and the C_CO_2__–O_OH_ bond for the bicarbonate–water system. The polarization direction of the oscillating electric field was then set along the same x direction. The choice of polarization direction was informed by Part I of this series [19], where static electric fields aligned with the molecular dipole moment and the regeneration-relevant C_CO_2__–N_MEA_ bond produced very similar results using an implicit-solvent model. In the present explicitly solvated systems, the total dipole moment of the cluster depends on the arrangement of the surrounding water molecules. Aligning the laser polarization with the regeneration-relevant bond therefore provides a consistent reference across independently prepared configurations. This alignment applies only to the initial configuration; the nuclear coordinates remain unconstrained during the subsequent Ehrenfest dynamics, allowing the molecules to move and reorient relative to the fixed laser-polarization direction. Nevertheless, the short simulation timescale does not provide complete orientational averaging, and a possible dependence of the observed mode selectivity on the initial molecular orientation cannot be ruled out. The excitation frequencies were selected to match the characteristic vibrational modes identified from the analysis described in Section 3.1. These included the C_CO_2__–N_MEA_ stretching modes of the zwitterion–water and carbamate–water systems; the symmetric and asymmetric C_CO_2__–O_CO_2__ stretching modes and the C_CO_2__–O_CO_2__–C_CO_2__ bending mode of all three systems; and the C_CO_2__–O_OH_ stretching mode of the bicarbonate–water system.

The vibrational modes selected for infrared excitation and their corresponding harmonic frequencies are summarized in Table 3. These frequencies were used to define the applied laser frequencies for the zwitterion–water, carbamate–water, and bicarbonate–water simulations. This frequency-selective approach was used to determine which vibrational modes most effectively transfer energy to the molecular systems and induce structural perturbations relevant to amine regeneration. It also enables assessment of vibrational mode selectivity in both the thermal and nonthermal responses of the CO_2_–amine absorption products. For completeness, time-dependent Ehrenfest electron–nuclear dynamics simulations were also performed in the presence of a static electric field, providing a direct dynamical counterpart to the static-field results reported in Part I [19].

For the pulsed-laser simulations, a sequence of short infrared pulses was applied to each molecular system. As illustrated in the upper panels of Figure 11, each pulse comprised three electric-field oscillation cycles, followed by an equal field-free relaxation interval. A total of 10 pulses was applied during each simulation, giving 30 laser-on oscillation cycles in total. This protocol was designed to introduce vibrational energy intermittently while allowing partial relaxation between successive excitation events, thereby enabling the investigation of transient nonequilibrium vibrational dynamics under repeated resonant excitation.

Continuous-wave (CW) infrared excitation was modelled using a single continuous signal comprising 30 electric-field oscillation cycles. The lower panels of Figure 11 show representative CW signals for the vibrational modes considered. In contrast to the pulsed laser protocol, CW excitation provides sustained resonant driving of the targeted modes and therefore enables assessment of the cumulative effects of continuous energy deposition and vibrational excitation. Together, the pulsed and CW simulations provide complementary insight into the response of the different CO_2_–amine systems to intermittent and sustained infrared excitation.

The infrared laser electric-field amplitudes investigated in this work ranged from 0.00 to 0.20 V/Å in increments of 0.02 V/Å. For clarity, Figure 11 shows representative signals corresponding to a field amplitude of 0.02 V/Å. All nonequilibrium Ehrenfest electron–nuclear dynamics simulations were performed without a thermostat, using an integration timestep of 0.005 fs and an initial temperature of 0 K. Unlike conventional NVE dynamics, the total energy of the molecular system is not expected to remain constant during laser excitation because the externally applied time-dependent electric field transfers energy to the system. Trajectories were sampled such that each electric-field oscillation cycle was represented by 100 snapshots, ensuring a consistent temporal resolution across all excitation frequencies. Each simulation condition was repeated ten times using independently selected configurations obtained from the equilibrated molecular dynamics trajectories and subsequently geometry optimized, and the reported results were averaged over these replicate trajectories. The relatively high field amplitudes were intentionally employed to compensate for the short timescales accessible to time-dependent Ehrenfest electron–nuclear dynamics simulations. Because the coupled electronic and nuclear dynamics can typically be propagated only over picosecond timescales, stronger fields are required to produce measurable nonequilibrium vibrational responses within computationally tractable trajectories. The field strengths considered here should therefore be regarded primarily as a means of probing the sensitivity of the molecular systems to mode-selective excitation rather than as direct representations of experimentally applied laser intensities [29,37].

The vibrational kinetic energies of the CO_2_–amine species and the surrounding water were obtained by post-processing the coordinate and velocity trajectories, together with the corresponding bond analysis and bond-energy outputs. Because DFTB+ does not directly report dynamical properties for selected subsets of atoms, the required subsystem-resolved quantities were calculated separately from the trajectories. Initiating all simulations at 0 K minimized contributions from pre-existing thermal motion, thereby isolating the laser-induced response and improving the signal-to-noise ratio of the statistical analysis. The vibrational kinetic energies of the CO_2_–amine species and solvent, as well as the average lengths, distribution widths, energies, and probabilities of elongated configurations of the regeneration-relevant bonds, were calculated from the first 12 electric-field oscillation cycles of the CW simulations, corresponding to the first four three-cycle excitation intervals.

## 4. Conclusions

This study investigated whether mode-selective infrared laser excitation could provide a molecular basis for less energy-demanding amine regeneration in post-combustion carbon capture. Nonequilibrium Ehrenfest electron–nuclear dynamics simulations of aqueous zwitterion, carbamate, and bicarbonate systems demonstrate that infrared laser energy deposition is strongly mode selective. Under both pulsed and continuous-wave infrared laser excitation, the C_CO_2__–O_CO_2__–C_CO_2__ bending mode provides the most consistently effective overall heating pathway across all three systems. However, strong total-system heating does not necessarily correspond to preferential deposition of vibrational kinetic energy into the CO_2_–amine species. The mode producing the strongest preferential energy deposition differs among the three absorption products: the symmetric C_CO_2__–O_CO_2__ stretch for the zwitterion, the C_CO_2__–N_MEA_ stretch for the carbamate, and the C_CO_2__–O_OH_ stretch for the bicarbonate. Conversely, the strongly heating C_CO_2__–O_CO_2__–C_CO_2__ bending mode preferentially deposits more energy into the surrounding water in the carbamate and bicarbonate systems. Infrared laser excitation can therefore control not only the magnitude of energy absorption, but also how that energy is partitioned between the reactive CO_2_–amine species and the solvent.

Infrared laser excitation also produces pronounced mode-dependent structural and energetic perturbations of the regeneration-relevant C_CO_2__–N_MEA_ and C_CO_2__–O_OH_ bonds. Selected modes increase their mean bond lengths and energies, broaden the corresponding structural and energetic distributions, and increase the probability of sampling transient elongated-bond configurations. The mode producing the strongest response is system dependent and is not necessarily the vibrational mode directly associated with the monitored bond. For the zwitterion, the C_CO_2__–O_CO_2__–C_CO_2__ bending and C_CO_2__–N_MEA_ stretching modes strongly perturb the C_CO_2__–N_MEA_ bond. For the carbamate, direct C_CO_2__–N_MEA_ excitation produces the strongest overall structural and energetic response, while the asymmetric C_CO_2__–O_CO_2__ stretch also produces substantial perturbations. For the bicarbonate, the symmetric C_CO_2__–O_CO_2__ stretch produces the largest increases in the mean C_CO_2__–O_OH_ bond length and its structural and energetic fluctuations, whereas the C_CO_2__–O_CO_2__–C_CO_2__ bending mode produces the largest increase in its mean bond energy. These findings are consistent with vibrational coupling and energy redistribution, through which excitation of one mode can influence other molecular motions associated with CO_2_ release.

The results identify three complementary effects through which infrared laser excitation may facilitate amine regeneration: efficient laser-induced heating, preferential energy deposition into the CO_2_–amine species rather than the surrounding solvent, and mode-selective structural and energetic perturbations of bonds involved in CO_2_ retention. No single vibrational mode maximizes all three effects across the investigated systems, indicating that selecting an excitation frequency is inherently a multi-objective problem. This response differs from the static-field behaviour reported in Part I, where the CO_2_ absorption products were stabilized and regeneration became energetically less favourable. Infrared laser excitation instead drives the systems away from equilibrium, producing thermal energy deposition alongside transient structural and energetic changes in regeneration-relevant bonds. Although these findings identify molecular mechanisms that may facilitate regeneration, they do not demonstrate a reduction in process-level energy consumption. Establishing such a benefit will require experimental validation and an assessment of the overall laser-assisted process efficiency, including laser capital and operating costs.

## Figures and Tables

**Table 1:** Selected bond lengths (Å) and bond angles (°) for representative water molecules and CO_2_–amine absorption products predicted using the MIO, 3OB, OB2, 3OB-FREQ, GFN1-xTB, and GFN2-xTB methods, together with the corresponding reference DFT values. The evaluated CO_2_–amine species are the MEA zwitterion, MEA carbamate, and TEA bicarbonate.

Parameter	3OB-FREQ	3OB	MIO	OB2	GFN1-xTB	GFN2-xTB	DFT [19]
Water							
O–H bond length	0.979	0.979	0.984	0.987	0.970	0.974	0.959
H–O–H bond angle	108.96	108.9	106.75	105.92	105.86	106.17	105.28
Zwitterion (MEA)							
C_CO_2__–N_MEA_ bond length	1.553	1.961	2.163	2.716	1.564	1.541	1.564
C_CO_2__–O_CO_2__ bond length	1.262	1.211	1.181	1.168	1.217	1.220	1.223
C_CO_2__–O_CO_2__–C_CO_2__ bond angle	129.64	146.53	160.52	178.77	133.7	131.09	134.06
Carbamate (MEA)							
C_CO_2__–N_MEA_ bond length	1.370	1.352	1.371	1.367	1.385	1.343	1.396
C_CO_2__–O_CO_2__ bond length	1.316	1.312	1.306	1.296	1.262	1.268	1.259
C_CO_2__–O_CO_2__–C_CO_2__ bond angle	119.32	119.27	121.59	121.38	124.04	120.83	125.65
Bicarbonate (TEA)							
C_CO_2__–O_OH_ bond length	1.347	1.347	1.347	1.347	1.347	1.347	1.372
C_CO_2__–O_CO_2__ bond length	1.293	1.296	1.282	1.281	1.252	1.247	1.246
C_CO_2__–O_CO_2__–C_CO_2__ bond angle	122.63	121.7	125.43	123.6	124.77	123.99	127.97

**Table 2:** Selected harmonic vibrational frequencies (cm^−1^) for representative water molecules and CO_2_–amine absorption products predicted using the MIO, 3OB, OB2, 3OB-FREQ, GFN1-xTB, and GFN2-xTB methods, together with the corresponding reference DFT values. The reported modes include characteristic stretching and bending vibrations of water, the MEA zwitterion, MEA carbamate, and TEA bicarbonate.

Parameter	3OB-FREQ	3OB	MIO	OB2	GFN1-xTB	GFN2-xTB	DFT [19]
Water							
O–H symmetric stretch	3653.47	3653.47	3711.82	3685.10	3675.22	3642.83	3853.46
O–H asymmetric stretch	3921.61	3921.61	3983.12	3877.76	3786.84	3650.88	3959.40
H–O–H bending	1381.00	1381.00	1524.91	1569.90	1438.07	1539.53	1649.24
Zwitterion (MEA)							
C_CO_2__–N_MEA_ stretch	872.19	686.97	912.24	1132.15	1117.21	1488.08	852.77
C_CO_2__–O_CO_2__ symmetric stretch	1283.17	1348.62	1298.75	1459.68	1345.57	1168.08	1337.38
C_CO_2__–O_CO_2__ asymmetric stretch	1629.19	1690.04	2154.74	2611.17	1662.23	1609.51	1781.77
C_CO_2__–O_CO_2__–C_CO_2__ bending	694.89	782.80	609.46	568.06	791.45	726.88	746.42
Carbamate (MEA)							
C_CO_2__–N_MEA_ stretch	1527.42	1693.95	1594.41	1587.48	1386.16	1489.71	1385.18
C_CO_2__–O_CO_2__ symmetric stretch	1179.20	1210.77	1181.52	1242.80	1167.39	1166.40	1146.88
C_CO_2__–O_CO_2__ asymmetric stretch	1439.00	1426.89	1392.36	1633.23	1588.59	1597.49	1566.01
C_CO_2__–O_CO_2__–C_CO_2__ bending	748.50	779.34	750.20	789.98	758.89	738.23	757.51
Bicarbonate (TEA)							
C_CO_2__–O_OH_ stretch	1092.86	1201.45	1040.46	1137.44	1090.00	1403.68	1145.88
C_CO_2__–O_CO_2__ symmetric stretch	1353.79	1416.91	1263.14	1462.13	1368.50	1073.13	1342.23
C_CO_2__–O_CO_2__ asymmetric stretch	1510.18	1544.74	1484.99	1715.98	1655.70	1692.62	1627.38
C_CO_2__–O_CO_2__–C_CO_2__ bending	721.96	738.52	735.72	730.35	707.76	751.65	704.14

**Table 3:** Vibrational modes selected for infrared laser excitation and their corresponding harmonic frequencies (cm^−1^) for the zwitterion–water, carbamate–water, and bicarbonate–water systems, calculated using the 3OB-FREQ parameter set. These frequencies were used to define the corresponding infrared laser excitation frequencies in the nonequilibrium Ehrenfest electron–nuclear dynamics simulations.

Mode	Zwitterion–Water	Carbamate–Water	Bicarbonate–Water
C_CO_2__–O_CO_2__–C_CO_2__ bending	694.89	748.50	721.96
C_CO_2__–O_CO_2__ symmetric stretch	1283.17	1179.20	1353.79
C_CO_2__–O_CO_2__ asymmetric stretch	1629.19	1439.00	1510.18
C_CO_2__–N_MEA_ stretch	872.19	1527.42	—
H_MEA_–N_MEA_ stretch	3323.49	3264.82	—
C_CO_2__–O_OH_ stretch	—	—	1092.86
H_OH_–O_OH_ stretch	—	—	2922.62

## Data Availability

Dataset available upon request from the corresponding author.

## Supplementary Materials

The following supporting information can be downloaded at https://www.mdpi.com/article/10.3390/molecules31193475/s1, Table S1: Bond-length distribution widths and absolute elongated-bond probabilities under static electric fields; Table S2: Bond-length distribution widths and absolute elongated-bond probabilities under resonant C_CO_2__–O_CO_2__–C_CO_2__ bending mode excitation; Table S3: Bond-length distribution widths and absolute elongated-bond probabilities under resonant C_CO_2__–N_MEA_ stretching mode excitation in the zwitterion–water and carbamate–water systems, and C_CO_2__–O_OH_ stretching mode excitation in the bicarbonate–water system; Table S4: Bond-length distribution widths and absolute elongated-bond probabilities under resonant symmetric C_CO_2__–O_CO_2__ stretch mode excitation; Table S5: Bond-length distribution widths and absolute elongated-bond probabilities under resonant asymmetric C_CO_2__–O_CO_2__ stretch mode excitation; Table S6: Bond-length distribution widths and absolute elongated-bond probabilities under resonant H_MEA_–N_MEA_ stretching mode excitation in the zwitterion–water and carbamate–water systems, and H_OH_–O_OH_ stretching mode excitation in the bicarbonate–water system.
