# Supplementary material for: Electric Field Effects on Amine Regeneration in Post-Combustion Carbon Capture—Part II: Mode-Selective Infrared Laser Excitation Probed by Nonequilibrium Ehrenfest Electron-Nuclear Dynamics
Source: Molecules. 2026 Sep 29;31(19):3475. doi: 10.3390/molecules31193475 (PMC13666188; doi:10.3390/molecules31193475)
Supplement: Supplementary file 1 [file molecules-31-03475-s001.zip › molecules-4533806-supplementary.pdf]

Supporting Information:

Electric Field Effects on Amine Regeneration in Post-Combustion Carbon Capture—Part II: Mode-Selective Infrared Laser Excitation Probed by Nonequilibrium Ehrenfest Electron-Nuclear Dynamics

Nasser D. Afify*, Xianfeng Fan and Martin B. Sweatman

Institute for Materials and Processes, School of Engineering, The University of Edinburgh, Edinburgh EH9 3FB, UK; x.fan@ed.ac.uk (X.F.); martin.sweatman@ed.ac.uk (M.B.S.)

*Correspondence: n.afify@ed.ac.uk

Note applicable to Tables S1–S6: The bond-length distribution width, σ, is the standard deviation of the sampled bond lengths within an individual trajectory. An elongated bond is defined operationally as a bond with length $r > \bar{r}_0 + 1.5\sigma_0$, where $\bar{r}_0$ and σ_0 are the mean bond length and standard deviation, respectively, calculated from the corresponding zero-field trajectory. The elongated-bond probability is the percentage of sampled bond lengths exceeding this threshold. Values are reported as the mean ± standard deviation across ten independent trajectories.

Table S1. Bond-length distribution widths and absolute elongated-bond probabilities under static electric fields.

Static electric field strength [V/Å]	Zwitterion–water C_{CO2}-N_{MEA} bond		Carbamate–water C_{CO2}-N_{MEA} bond		Bicarbonate–water C_{CO2}-O_{OH} bond	
	Bond length distribution width σ [Å]	Elongated bond probability [%]	Bond length distribution width σ [Å]	Elongated bond probability [%]	Bond length distribution width σ [Å]	Elongated bond probability [%]
0.00	0.00702 ± 0.00047	6.59 ± 0.49	0.00500 ± 0.00021	6.05 ± 0.46	0.00644 ± 0.00046	6.22 ± 0.37
0.02	0.00697 ± 0.00046	6.61 ± 0.49	0.00503 ± 0.00022	5.93 ± 0.49	0.00598 ± 0.00027	6.14 ± 0.38
0.04	0.00697 ± 0.00046	6.68 ± 0.51	0.00500 ± 0.00021	6.09 ± 0.47	0.00598 ± 0.00027	6.14 ± 0.38
0.06	0.00697 ± 0.00046	6.70 ± 0.51	0.00500 ± 0.00021	6.12 ± 0.46	0.00598 ± 0.00027	6.15 ± 0.39
0.08	0.00697 ± 0.00046	6.71 ± 0.51	0.00500 ± 0.00021	6.13 ± 0.47	0.00599 ± 0.00027	6.16 ± 0.38
0.10	0.00697 ± 0.00046	6.72 ± 0.50	0.00500 ± 0.00021	6.28 ± 0.45	0.00599 ± 0.00027	6.19 ± 0.38
0.12	0.00663 ± 0.00053	6.88 ± 0.33	0.00500 ± 0.00021	6.16 ± 0.46	0.00599 ± 0.00027	6.20 ± 0.38
0.14	0.00610 ± 0.00042	7.03 ± 0.52	0.00501 ± 0.00021	6.19 ± 0.45	0.00599 ± 0.00027	6.22 ± 0.38
0.16	0.00697 ± 0.00046	6.66 ± 0.49	0.00501 ± 0.00021	6.24 ± 0.45	0.00600 ± 0.00027	6.26 ± 0.37
0.18	0.00697 ± 0.00046	6.73 ± 0.50	0.00501 ± 0.00021	6.28 ± 0.45	0.00600 ± 0.00027	6.29 ± 0.36
0.20	0.00697 ± 0.00046	6.72 ± 0.50	0.00501 ± 0.00021	6.33 ± 0.46	0.00600 ± 0.00027	6.30 ± 0.36

Table S2. Bond-length distribution widths and absolute elongated-bond probabilities under resonant C_{CO_2}- O_{CO_2}- C_{CO_2} bending mode excitation.

Laser electric field strength [V/Å]	Zwitterion–water C_{CO2}-N_{MEA} bond		Carbamate–water C_{CO2}-N_{MEA} bond		Bicarbonate–water C_{CO2}-O_{OH} bond	
	Bond length distribution width σ [Å]	Elongated bond probability [%]	Bond length distribution width σ [Å]	Elongated bond probability [%]	Bond length distribution width σ [Å]	Elongated bond probability [%]
0.00	0.00812 ± 0.00055	7.45 ± 0.39	0.00481 ± 0.00024	6.39 ± 0.38	0.00588 ± 0.00025	6.22 ± 0.35
0.02	0.01000 ± 0.00071	12.9 ± 1.1	0.00548 ± 0.00023	10.2 ± 1.1	0.00615 ± 0.00018	8.03 ± 0.52
0.04	0.0124 ± 0.0012	19.0 ± 2.4	0.00684 ± 0.00030	14.6 ± 1.5	0.00726 ± 0.00037	13.2 ± 1.6
0.06	0.0177 ± 0.0014	27.9 ± 2.4	0.00843 ± 0.00052	21.4 ± 1.5	0.00954 ± 0.00069	20.5 ± 1.8
0.08	0.0227 ± 0.0017	33.1 ± 2.3	0.01012 ± 0.00073	26.7 ± 1.5	0.0114 ± 0.0012	24.8 ± 2.4
0.10	0.0284 ± 0.0018	39.6 ± 2.1	0.01174 ± 0.00089	31.3 ± 1.0	0.0147 ± 0.0012	32.8 ± 1.6
0.12	0.0345 ± 0.0015	44.3 ± 1.5	0.0134 ± 0.0011	35.33 ± 0.73	0.0178 ± 0.0014	37.4 ± 1.4
0.14	0.0411 ± 0.0015	47.52 ± 0.82	0.0152 ± 0.0013	37.93 ± 0.93	0.0217 ± 0.0016	41.2 ± 1.5
0.16	0.0466 ± 0.0020	51.43 ± 0.73	0.0178 ± 0.0016	41.1 ± 1.0	0.0261 ± 0.0019	44.9 ± 1.4
0.18	0.0545 ± 0.0031	53.25 ± 0.70	0.0192 ± 0.0017	44.0 ± 1.2	0.0286 ± 0.0018	47.3 ± 1.5
0.20	0.0590 ± 0.0048	54.79 ± 0.86	0.0216 ± 0.0020	45.88 ± 0.99	0.0322 ± 0.0018	49.8 ± 1.1

Table S3. Bond-length distribution widths and absolute elongated-bond probabilities under resonant C_{CO2}-N_{MEA} stretching mode excitation in the zwitterion–water and carbamate–water systems, and C_{CO_2}-O_{OH} stretching mode excitation in the bicarbonate–water system.

Laser electric field strength [V/Å]	Zwitterion–water C_{CO2}-N_{MEA} bond		Carbamate–water C_{CO2}-N_{MEA} bond		Bicarbonate–water C_{CO2}-O_{OH} bond	
	Bond length distribution width σ [Å]	Elongated bond probability [%]	Bond length distribution width σ [Å]	Elongated bond probability [%]	Bond length distribution width σ [Å]	Elongated bond probability [%]
0.00	0.00756 ± 0.00053	7.04 ± 0.45	0.00503 ± 0.00019	6.35 ± 0.45	0.00596 ± 0.00024	6.38 ± 0.40
0.02	0.00996 ± 0.00077	14.1 ± 1.6	0.00783 ± 0.00026	15.71 ± 0.81	0.00691 ± 0.00053	10.6 ± 1.2
0.04	0.0150 ± 0.0016	22.1 ± 2.3	0.01726 ± 0.00041	30.72 ± 0.82	0.00883 ± 0.00094	16.0 ± 2.0
0.06	0.0209 ± 0.0025	28.8 ± 2.7	0.02696 ± 0.00062	37.90 ± 0.57	0.0119 ± 0.0013	24.0 ± 1.5
0.08	0.0270 ± 0.0036	35.9 ± 1.8	0.03674 ± 0.00085	41.62 ± 0.59	0.0150 ± 0.0017	29.9 ± 1.9
0.10	0.0332 ± 0.0048	40.8 ± 1.4	0.0464 ± 0.0011	44.20 ± 0.41	0.0184 ± 0.0022	34.6 ± 2.1
0.12	0.0393 ± 0.0057	44.0 ± 1.1	0.0557 ± 0.0014	45.92 ± 0.32	0.0221 ± 0.0027	35.5 ± 3.7
0.14	0.0452 ± 0.0062	46.3 ± 1.1	0.0646 ± 0.0017	47.08 ± 0.30	0.0263 ± 0.0035	40.9 ± 1.8
0.16	0.0518 ± 0.0064	48.2 ± 1.0	0.0729 ± 0.0021	48.03 ± 0.29	0.0330 ± 0.0051	42.9 ± 1.6
0.18	0.0583 ± 0.0063	49.9 ± 1.1	0.0805 ± 0.0024	48.90 ± 0.27	0.0359 ± 0.0055	44.6 ± 1.6
0.20	0.0611 ± 0.0082	47.5 ± 4.2	0.0874 ± 0.0026	49.59 ± 0.26	0.0411 ± 0.0066	46.1 ± 1.5

Table S4. Bond-length distribution widths and absolute elongated-bond probabilities under resonant symmetric C_{CO_2}- O_{CO_2} stretch mode excitation.

Laser electric field strength [V/Å]	Zwitterion–water C_{CO2}-N_{MEA} bond		Carbamate–water C_{CO2}-N_{MEA} bond		Bicarbonate–water C_{CO2}-O_{OH} bond	
	Bond length distribution width σ [Å]	Elongated bond probability [%]	Bond length distribution width σ [Å]	Elongated bond probability [%]	Bond length distribution width σ [Å]	Elongated bond probability [%]
0.00	0.00689 ± 0.00053	6.94 ± 0.48	0.00501 ± 0.00024	6.03 ± 0.45	0.00600 ± 0.00027	6.37 ± 0.36
0.02	0.00929 ± 0.00039	14.3 ± 1.0	0.00599 ± 0.00022	10.83 ± 0.83	0.01060 ± 0.00080	20.2 ± 1.9
0.04	0.01261 ± 0.00053	20.7 ± 1.6	0.00800 ± 0.00026	18.3 ± 1.4	0.0166 ± 0.0016	31.9 ± 1.9
0.06	0.01622 ± 0.00077	26.2 ± 1.7	0.01053 ± 0.00038	25.8 ± 1.5	0.0235 ± 0.0024	37.4 ± 1.6
0.08	0.0200 ± 0.0010	31.5 ± 1.7	0.01330 ± 0.00056	31.5 ± 1.3	0.0303 ± 0.0032	40.7 ± 1.2
0.10	0.0239 ± 0.0014	35.7 ± 1.4	0.01626 ± 0.00077	35.7 ± 1.2	0.0370 ± 0.0039	43.1 ± 1.1
0.12	0.0278 ± 0.0017	38.4 ± 1.3	0.0193 ± 0.0010	38.83 ± 0.93	0.0432 ± 0.0046	44.7 ± 1.1
0.14	0.0316 ± 0.0020	40.6 ± 1.1	0.0226 ± 0.0014	41.31 ± 0.70	0.0489 ± 0.0051	46.1 ± 1.1
0.16	0.0352 ± 0.0023	38.3 ± 3.4	0.0260 ± 0.0018	42.79 ± 0.57	0.0538 ± 0.0054	47.40 ± 0.90
0.18	0.0387 ± 0.0025	43.52 ± 0.88	0.0274 ± 0.0033	40.4 ± 3.5	0.0581 ± 0.0055	48.36 ± 0.83
0.20	0.0422 ± 0.0028	44.58 ± 0.80	0.0330 ± 0.0026	45.08 ± 0.44	0.0618 ± 0.0056	49.04 ± 0.78

Table S5. Bond-length distribution widths and absolute elongated-bond probabilities under resonant asymmetric C_{CO_2}- O_{CO_2} stretch mode excitation.

Laser electric field strength [V/Å]	Zwitterion–water C_{CO2}-N_{MEA} bond		Carbamate–water C_{CO2}-N_{MEA} bond		Bicarbonate–water C_{CO2}-O_{OH} bond	
	Bond length distribution width σ [Å]	Elongated bond probability [%]	Bond length distribution width σ [Å]	Elongated bond probability [%]	Bond length distribution width σ [Å]	Elongated bond probability [%]
0.00	0.00640 ± 0.00041	6.06 ± 0.69	0.00505 ± 0.00020	6.30 ± 0.47	0.00607 ± 0.00029	6.32 ± 0.43
0.02	0.00640 ± 0.00042	6.15 ± 0.55	0.00752 ± 0.00037	15.9 ± 1.5	0.00720 ± 0.00032	11.82 ± 0.90
0.04	0.00658 ± 0.00041	6.48 ± 0.53	0.01277 ± 0.00068	31.15 ± 0.80	0.01000 ± 0.00033	21.1 ± 1.4
0.06	0.00695 ± 0.00041	8.06 ± 0.69	0.01880 ± 0.00098	37.18 ± 0.61	0.01337 ± 0.00055	26.1 ± 2.6
0.08	0.0093 ± 0.0018	13.3 ± 3.3	0.0238 ± 0.0022	37.1 ± 2.8	0.01687 ± 0.00082	33.1 ± 1.4
0.10	0.00818 ± 0.00039	13.3 ± 1.2	0.0326 ± 0.0015	43.77 ± 0.35	0.0205 ± 0.0011	36.4 ± 1.3
0.12	0.00897 ± 0.00039	16.2 ± 1.1	0.0405 ± 0.0016	45.59 ± 0.26	0.0241 ± 0.0014	38.9 ± 1.1
0.14	0.00982 ± 0.00039	18.6 ± 1.1	0.0493 ± 0.0018	46.94 ± 0.24	0.0276 ± 0.0016	40.95 ± 0.94
0.16	0.01071 ± 0.00040	20.8 ± 1.0	0.0590 ± 0.0019	48.17 ± 0.22	0.0310 ± 0.0019	42.37 ± 0.84
0.18	0.01164 ± 0.00041	22.38 ± 0.97	0.0696 ± 0.0018	49.11 ± 0.21	0.0343 ± 0.0021	43.46 ± 0.75
0.20	0.01261 ± 0.00042	24.05 ± 0.93	0.0807 ± 0.0017	50.11 ± 0.21	0.0375 ± 0.0023	44.43 ± 0.73

Table S6. Bond-length distribution widths and absolute elongated-bond probabilities under resonant H_{MEA}-N_{MEA} stretching mode excitation in the zwitterion–water and carbamate–water systems, and H_{OH}-O_{OH} stretching mode excitation in the bicarbonate–water system.

Laser electric field strength [V/Å]	Zwitterion–water C_{CO2}-N_{MEA} bond		Carbamate–water C_{CO2}-N_{MEA} bond		Bicarbonate–water C_{CO2}-O_{OH} bond	
	Bond length distribution width σ [Å]	Elongated bond probability [%]	Bond length distribution width σ [Å]	Elongated bond probability [%]	Bond length distribution width σ [Å]	Elongated bond probability [%]
0.00	0.00624 ± 0.00044	4.78 ± 0.65	0.00492 ± 0.00019	6.29 ± 0.45	0.00607 ± 0.00036	5.58 ± 0.45
0.02	0.00560 ± 0.00037	3.91 ± 0.71	0.00485 ± 0.00022	5.50 ± 0.60	0.00614 ± 0.00038	6.05 ± 0.56
0.04	0.00557 ± 0.00038	3.49 ± 0.77	0.00476 ± 0.00021	4.86 ± 0.67	0.00623 ± 0.00040	6.62 ± 0.82
0.06	0.00556 ± 0.00038	3.34 ± 0.75	0.00469 ± 0.00020	4.42 ± 0.71	0.00605 ± 0.00043	6.6 ± 1.0
0.08	0.00556 ± 0.00039	2.75 ± 0.70	0.00466 ± 0.00019	3.83 ± 0.77	0.00642 ± 0.00044	7.0 ± 1.1
0.10	0.00557 ± 0.00040	2.42 ± 0.73	0.00465 ± 0.00018	3.62 ± 0.75	0.00654 ± 0.00045	7.4 ± 1.2
0.12	0.00563 ± 0.00040	2.31 ± 0.69	0.00468 ± 0.00017	3.40 ± 0.71	0.00668 ± 0.00046	7.9 ± 1.2
0.14	0.00570 ± 0.00040	1.93 ± 0.46	0.00472 ± 0.00017	3.23 ± 0.66	0.00683 ± 0.00047	9.0 ± 1.0
0.16	0.00579 ± 0.00039	1.77 ± 0.45	0.00517 ± 0.00038	3.93 ± 0.94	0.00701 ± 0.00048	9.76 ± 0.98
0.18	0.00591 ± 0.00038	1.78 ± 0.46	0.00493 ± 0.00016	3.21 ± 0.58	0.00722 ± 0.00050	10.42 ± 0.97
0.20	0.00606 ± 0.00037	1.77 ± 0.49	0.00510 ± 0.00016	3.31 ± 0.55	0.00746 ± 0.00050	11.17 ± 0.85
